# Replication Rate, Framing, and Format Affect Attitudes and Decisions about Science Claims

**DOI:** 10.3389/fpsyg.2016.01826

**Published:** 2016-11-22

**Authors:** Ralph M. Barnes, Stephanie J. Tobin, Heather M. Johnston, Noah MacKenzie, Chelsea M. Taglang

**Affiliations:** ^1^Department of Psychology, Montana State UniversityBozeman, MT, USA; ^2^School of Psychology, Australian Catholic UniversityBanyo, QLD, Australia; ^3^Psychology Department, Columbus State Community CollegeColumbus, OH, USA; ^4^Department of Social Sciences, Clermont College, University of CincinnatiBatavia, OH, USA; ^5^Department of Psychology and Counseling, Hood CollegeFrederick, MD, USA

**Keywords:** framing, natural frequencies, probability judgments, public perception of science, replication, representation of information

## Abstract

A series of five experiments examined how the evaluation of a scientific finding was influenced by information about the number of studies that had successfully replicated the initial finding. The experiments also tested the impact of frame (negative, positive) and numeric format (percentage, natural frequency) on the evaluation of scientific findings. In Experiments 1 through 4, an attitude difference score served as the dependent measure, while a measure of choice served as the dependent measure in Experiment 5. Results from a diverse sample of 188 non-institutionalized U.S. adults (Experiment 2) and 730 undergraduate college students (Experiments 1, 3, and 4) indicated that attitudes became more positive as the replication rate increased and attitudes were more positive when the replication information was framed positively. The results also indicate that the manner in which replication rate was framed had a greater impact on attitude than the replication rate itself. The large effect for frame was attenuated somewhat when information about replication was presented in the form of natural frequencies rather than percentages. A fifth study employing 662 undergraduate college students in a task in which choice served as the dependent measure confirmed the framing effect and replicated the replication rate effect in the positive frame condition, but provided no evidence that the use of natural frequencies diminished the effect.

## Introduction

“The gold standard for reliability is independent replication.”

–Frank and Saxe (2012).

“Even 100 failures in a row to replicate, following statistical significance, will not impugn the original decision to reject H_0_ and affirm H_1_.”

–Sohn (1998).

Science journalists may announce that a particular substance causes cancer, only to announce a few weeks or months later that subsequent studies failed to confirm the earlier finding. The public may be informed that a new drug treats a particular disorder safely, only to be told that further research indicates that the drug is either not safe or not effective (or both). After the findings of an initial study have been made public, science journalists might report that subsequent studies have replicated or, more likely, failed to replicate the initial results. How are members of the general public, who are regularly bombarded with conflicting scientific reports, influenced by this information? This paper will present several studies that investigate how information about research replication rates (the percentage of attempted replications of a study that succeeded in replicating the study) impact non-scientists' attitudes about science claims. Furthermore, the current studies investigate how the format in which replication rate information is presented can influence attitudes.

Many scientists feel that replication is a critical component of science (e.g., Ioannidis and Khoury, 2011; Santer et al., 2011; Tomasello and Call, 2011). Commonly touted benefits of replication include the ability to detect bias and random error (Bayarri and Berger, 1991), the ability to eliminate investigator error (Cohen, 1997) and Type 1 error as explanations for research results, and the creation of a cumulative body of work (Fowler, 1995). The importance of replication has been emphasized by some as a reaction to claims that a large proportion of scientific findings, are false positives (Ioannidis, 2005; see also Risch, 2000; Sterne and Davey Smith, 2001; Hirschhorn et al., 2002; Wacholder et al., 2004; Simmons et al., 2011).

The concept of replication is discussed in the mass media and information regarding replications (and failed replications) therefore has the potential to influence popular opinion regarding scientific claims. For instance, the media informed the general public about failures to replicate Pons and Fleischmann's cold fusion results (Browne, 1989), Andrew Wakefield's autism results (Boseley, 2010), Benveniste's water memory results (Sullivan, 1988), and the finding that the XMRV retrovirus was associated with chronic fatigue syndrome (Tuller, 2011). A widely disseminated New Yorker article aimed at a non-scientist audience (Leher, 2010) frankly addressed issues such as replicability and the file drawer effect.

The present studies were not designed to answer the question of how (or whether) confidence in a finding *should* be modified based on the outcome of replication efforts. Rather the present goal is descriptive: we wish to find out how knowledge about replication efforts affects the manner in which non-scientists alter their confidence in research findings. Many non-scientists lack a firm grasp of scientific and research methods, so we have no clear expectation of the amount of influence replication information will have on lay persons. However, we can generate the more conservative hypothesis that non-scientists are impacted by knowledge of replication in such a way that knowledge of a failed replication of a study will cause lay persons to reduce their confidence in that study.

When people make decisions under conditions of uncertainty, the framing of the information can influence their decisions (Tversky and Kahneman, 1981; Levin et al., 1998; Gong et al., 2013). Broadly speaking, framing effects are revealed when the wording used to describe a given piece of information has an impact on the choices and decisions individuals make. Framing effects have been demonstrated to affect choice, attitude, and behavior in a wide range of tasks, but not all framing studies explore the same phenomenon. Levin et al. (1998) include three types of framing in their typology: risky choice, goal, and attribute. In attribute framing, the dependent measure of interest is the evaluation of a single option (e.g., whether-or-not a claim is true) rather than a choice between independent options (e.g., whether-or-not one option is preferable to another), therefore the framing paradigm used in the present studies is an example of attribute framing. A consistent finding in the attribute framing literature is that a particular alternative is rated more favorably when described positively than when described negatively (Davis and Bobko, 1986).

In the attribute framing literature (see Sher and McKenzie, 2006 for a review), researchers sometimes claim that two different frames contain logically equivalent information (i.e., contain the same logical content). Cases where both frames contain logically equivalent information may not be equivalent in other crucial aspects, however. Some of the mechanisms proposed to explain attribute framing are concerned with the ways that differently framed statements are nonequivalent. The first possibility (Sher and McKenzie, 2006) is that two different logically equivalent frames may be informationally inequivalent because of information leakage: the communicator's choice of frame may contain information (e.g., the choice that the communicator favors) and this leaked information may be detected by the recipient of the communication (McKenzie and Nelson, 2003; Sher and McKenzie, 2006). The second possibility is that people interpret “60% replication rate” to mean “at least 60% have succeeded in replicating” and interpret “40% failure to replicate” to mean “at least 40% have failed to replicate” (Macdonald, 1986; Mandel, 2001). A third explanation of the attribute framing effect relies on the notion that people are sensitive to the descriptive valence of the words employed. Levin and Gaeth (1988) have argued that an attribute described with a positive label evokes favorable associations in memory, while an attribute described with a negative label evokes unfavorable associations in memory.

Not only do those who intend to communicate replication rate information to others have a choice of frame (positive, negative) they also have a choice of numeric format. The claim “10 studies attempted to replicate finding X, and 6 succeeded” is presented in terms of natural frequencies (Hoffrage et al., 2002). Another important characteristic of natural frequencies is that the total sample size and number of cases in a given subset are both transparent. When the same information is converted to probability (0.6) or percentage (60%), the number of cases in the subset and the total sample size are no longer available. So, proportion and percentage retain only one out of three piece of information found in the natural frequencies.

Natural frequencies have been compared with probabilities in the context of the debate concerning Bayesian reasoning (Gigerenzer, 1996; Kahneman and Tversky, 1996). Some have claimed that, due to their evolutionary heritage, humans have a natural predisposition to think in terms of natural frequencies (Gigerenzer and Hoffrage, 1995; Cosmides and Tooby, 1996; Brase et al., 1998; Hoffrage et al., 2000). Replacing probabilities with natural frequencies tends to minimize a number of biases (Fiedler, 1988; Gigerenzer et al., 1991; Koehler et al., 1994). Slovic et al. (2000) found that risk estimates were impacted by numeric format (probabilities vs. natural frequencies) and Purchase and Slovic (1999) found that when risks are small, presenting them in terms of frequency rather than proportion or percent has a tendency to alarm people. Regardless of the theoretical value of the manipulation, there is a simple practical reason for presenting participants with probability information in both percentage and natural frequency formats: if the format that probability information is presented in interacts with information about replication rate, then knowledge of this effect would be of use to producers and consumers of science information.

Note that in the English language one generally does not substitute “no percent” for “0%” or “all percent” for “100%.” When using natural frequencies, however, an individual sometimes has the option to use either Arabic numerals or verbal descriptors to specify the quantity of a subcategory. For instance, a particular situation could be described with the phrase “0 of the 5 studies replicated the effect” or the phrase “none of the 5 studies replicated the effect.” These two phrases are mathematically (and informationally) equivalent, so one might predict that they would be psychologically equivalent as well. As Feynman (1967) has pointed out, however, mathematical equivalence is not the same as psychological equivalence. Somewhat related to this issue, there is extensive research exploring the unique aspects of qualitative probability expressions and quantitative probability values (Toogood, 1980; Beyth-Marom, 1982; Nakao and Axelrod, 1983; Budescu and Wallsten, 1985; Mosteller and Youtz, 1990). Qualitative probability expressions include such verbal expressions as mostly, very likely, exceptionally unlikely, and rare while quantitative probability values include such numeric expressions as 90% likelihood and 1% likelihood. Though this literature reveals that these two manners of presenting probability information are not always interpreted the same way by participants, it should be noted that, unlike the natural frequency phrases given as an example above, qualitative probability expressions are neither logically nor mathematically equivalent to quantitative probability values. Since Arabic numbers might have different psychological effects than the use of qualitative terms, and both are found in media reports on science, we will use both kinds of expressions in the current study.

One of our major goals concerns a practical application; we would like to determine how high the replication rate of a study has to be before the knowledge about the replication rate will actually cause an increase, rather than a decrease, in confidence about that finding. Our second major goal is to determine if replication rate affects participants in a way that is independent of numeric format. Our third major goal is to determine what effect, if any, the framing of replication rate information has on attitudes. We have chosen attitude difference as the dependent measure with which to achieve our research goals.

Our first hypothesis (H1) is that participants will be sensitive to replication rate information. In the absence of a compelling theoretical reason to expect a more complex geometrical trend, it is our hypothesis that the relationship between attitude difference scores and replication rate will be linear: higher replication rates will be associated with more favorable attitudes toward the claims. Given the findings reported in studies that employed an attribute framing paradigm, our second hypothesis (H2) is that the framing of the replication rate information will have at least as strong an impact as the replication rate information itself. More specifically, it is our hypothesis that positively framed replication rates will lead to more favorable attitudes than negatively framed replication rates. Our third hypothesis (H3) is that the framing effect will be less pronounced when the numeric format of replication rate information is in natural frequencies rather than percentages. The third hypothesis is based on research that indicates that human reasoning is facilitated by the use of natural frequencies. Finally, it is our hypothesis (H4) that when replication rate information is presented as natural frequencies, the attitude difference will not depend on whether the natural frequency is presented with Arabic numerals (e.g., 0 out of 10) or words (e.g., none of the 10).

## Experiment 1a

The goal of Experiment 1 was to test our first two hypotheses using replication rate information in percentages. Thus, replication rate information was presented in terms of percentages and was framed in either a positive or negative fashion. We predicted that increasingly larger replication rates would be associated with more positive attitudes and that this would be evidenced by a significant linear trend, with no other significant trend components. We also predicted that negative frames would lead to more negative attitudes than would positive frames and the effect size associated with the framing effect would be at least moderate in size.

### Methods

#### Ethics statement

Ethics approval for Studies 1 through 5 was obtained from all institutions at which data was collected. These institutions include Columbus State Community College (CSCC), Haverford College (HC), Lafayette College (LC), Montana State University (MSU), Salem State University (SSU), University of Cincinnati-Clermont College (UCC), and The University of Houston (UH). For Study 1, data was collected and IRB approval was granted from CSCC, HC, SSU, and UH. For Study 2, IRB approval was granted from Lafayette College. For Studies 3 and 4, data was collected, and IRB approval was granted from CSCC, LC, and UCC. For Study 5, data was collected, and IRB approval was granted from MSU and UCC.

In all cases a written information sheet or a computer screen containing the information was provided to participants, but the committees waived the need for written informed consent from the participants as the research was low risk. Some minor deception was necessary to test our hypotheses, participants' rights and welfare were not adversely affected, and a full debriefing was provided at the end of the study.

#### Participants

One hundred and eighty-three undergraduate students enrolled in psychology courses volunteered to participate. Data from 11 participants were excluded because of failure to answer all the items in the questionnaire and/or failure to follow instructions. Seventy-eight percent of the remaining 172 participants were female. The age range for the sample was 18–49 and the average age was 21.9. Seven participants were students at a private liberal arts college, 119 were enrolled at a private university, 11 were enrolled at a state college, and 35 were enrolled at a community college. All participants were given extra-credit in their psychology courses as compensation. The stimuli, procedure, and primary dependent measure employed in Experiments 1 through 4 were novel. For this reason, we did not know what effect sizes would be likely to obtain. We therefore aimed to recruit 100 participants per frame for each experiment. Due to incomplete questionnaires and failure to follow instructions the exact number of participants per was never exactly 100. However, in all studies the decisions to terminate data collection were always made prior to looking at any of the data.

#### Stimuli and procedure

Stimuli consisted of 24 science claims about topics that were either fictions created by the authors or obscure topics that would be unfamiliar to participants. Thus, the claims were designed to function as the “blank predicates” that are commonly used by psychologists interested in deductive reasoning. Twelve of the claims were critical to this study (see Table S1) and the other 12 were distractor items that were included to keep participants from determining the purpose of the study. The stimuli for all experiments have been included in the Supplementary Materials.

Additional information for the 12 critical items consisted of the replication rate of studies that supported each initial claim (see Tables S2, S3). The additional information was framed in either a positive or negative fashion (a between-subjects manipulation). Frame was chosen as a between-subjects variable because we worried that if items on a given questionnaire switched between positively and negatively framed information, participants might be more likely to misread an item, and give a response appropriate to the wrong frame. In the positive frame condition, six replication rates (percentages of studies that successfully replicated the results of an earlier study) were chosen (9, 17, 24, 69, 77, and 84%); creating six potential versions of additional information for each initial claim. In the negative frame condition, six replication failure rates (percentages of studies that failed to replicate the results of an earlier study) were used (91, 83, 76, 31, 23, and 16%) creating six potential versions of additional information for each initial claim. For a given frame (positive, negative) and initial claim, the six related pieces of additional information differed only in regards to the percentage of successful replications reported. The additional information created for distractor items did not include any information regarding the replication (or lack thereof) of scientific studies. Distractor items consisted of science claims that contained additional supporting information that was unrelated to replication rate.

Each participant was randomly assigned to complete 1 of 16 (8 positive framed, 8 negatively framed) paper and pencil questionnaire variants (see Stimuli S1 for an example). Each questionnaire variant contained 24 science claims: 12 distractor items and 12 critical items. Six of the critical items were science claims in isolation (baseline items) and six of the critical items were science claims paired with additional information. For each critical item, a questionnaire would include either the baseline version of the item or the claim paired with additional information but never both. As with the critical items, 6 of the distractor items were science claims in isolation and 6 of the distractor items were science claims paired with additional information. Half of the questionnaire variants contained replication rate presented in a positive frame and half contained replication rate presented in a negative frame. In each questionnaire the initial section of 24 science claims was followed by several demographic questions.

The sequence of the 24 claims was randomized with the constraint that no more than three distractor items or three critical items could appear in a row. Additionally, no more than two non-baseline items could appear in a row. Pairings of critical items and replication rates was unique to each questionnaire. For instance, if a 17% replication rate was matched to science claim number 1 in variant 8, then none of the other variants would pair science claim number 1 with that particular replication rate.

After each science claim, respondents were presented with an opportunity to indicate their attitude toward the claim using a 6-point scale that ranged from strongly favor (1) to strongly oppose (6). It was unlikely that participants would find each of the critical science claims equally compelling, therefore difference scores, rather than raw attitude scores, served as the dependent measure. Had we used raw attitude scores, we would not have been able to determine the unique contributions of plausibility of the claim and information about replication rate. We used the responses to the baseline items (critical items not paired with additional information) to calculate mean attitude scores for each of the 12 claims. For each critical item paired with additional replication rate information trial, the attitude score was subtracted from the mean score of the appropriate critical claim in isolation (the mean baseline attitude score). A negative difference scores indicates that participants found a science claim to be less convincing when it was followed by additional information. For instance, across all participants presented with the baseline version of claim number 10, the average attitude was 3.1. If a participant responded with a “4” on the 1–6 scale for claim 10 on a trial in which the claim 10 was followed by additional information about the percentage of studies that successfully replicated the research upon which the claim was based, then that participant's difference score would be −0.9, indicating a shift away from the average baseline attitude of nearly one integer on the 6 point scale.

In this experiment, and all subsequent experiments, decisions to terminate data collection were made before any data were looked at. All of our studies, pilot studies, and conditions related to the topic of replication rate have been reported. Over the course of the current paper, all independent, and dependent variables have been fully reported. That is, the authors have no additional studies or data related to this topic in a “file drawer.”

### Results and discussion

**Figure 1:**
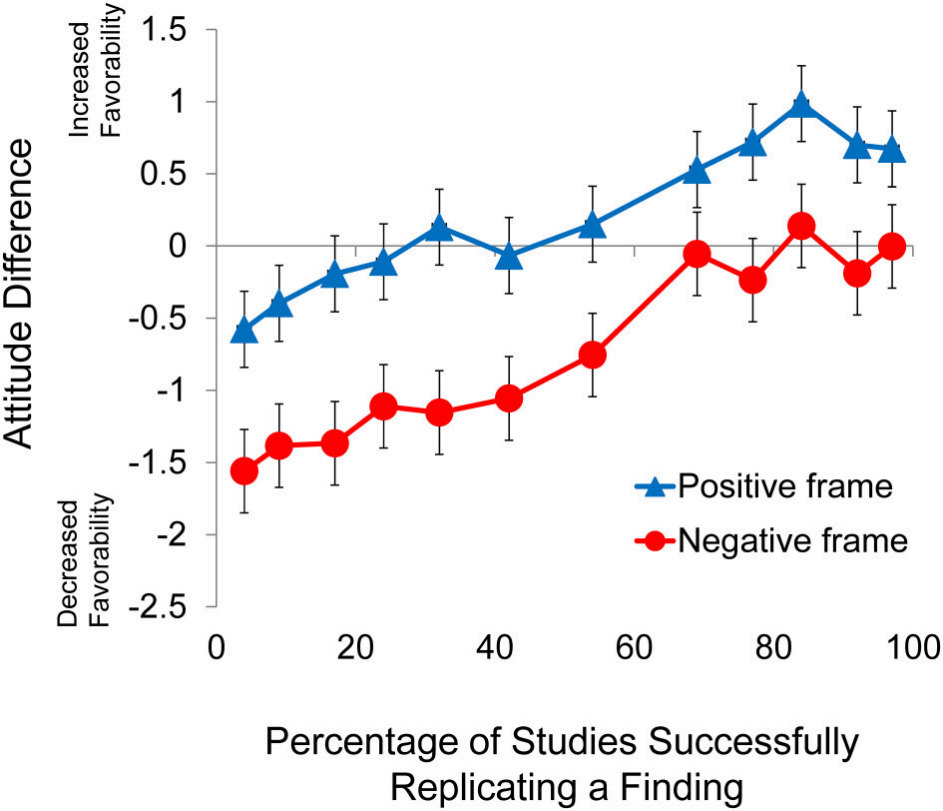
**Difference score as a function of frame for Experiments 1a and 1b**. Error bars indicate 95% confidence intervals.

Descriptive statistics for Experiment 1a are presented in Table 1 and the pattern of data for Experiment 1a is also presented graphically in Figure 1 (Data Available from Montana State University Scholarworks, https://doi.org/10.15788/M23014). Preliminary analyses considering gender and age were conducted on this experiment (and also Experiments 1b, 2, 3, and 4) revealed no significant effects involving either gender or age (*p* > 0.19 across Experiments 1a, 1b, 2, 3, and 4). Due to these findings, and because there were no theoretical reasons to expect that the replication rate or framing effects would be influenced by these variables, all reported analyses involve data in aggregate form.

**Table 1:** **Mean and standard deviation of difference scores for Experiments 1a and 1b**.

**Experiment**	**Replication rate (%)**	**Positive frame *M(SD)***	**Negative frame *M(SD)***
1b	4	−0.577 (1.38)^*^	−1.560 (1.33)^*^
1a	9	−0.397 (1.33)^*^	−1.384 (1.39)^*^
1a	17	−0.193 (1.36)	−1.367 (1.25)^*^
1a	24	−0.109 (1.33)	−1.111 (1.39)^*^
1b	32	0.131 (1.30)	−1.154 (1.07)^*^
1b	42	−0.066 (1.25)	−1.056 (1.21)^*^
1b	54	0.151 (1.38)	−0.755 (1.24)
1a	69	0.529 (1.34)^*^	−0.055 (1.20)
1a	77	0.720 (1.50)^*^	−0.236 (1.30)
1a	84	0.986 (1.19)^*^	0.139 (1.37)
1b	92	0.700 (1.46)^*^	−0.189 (1.36)
1b	97	0.673 (1.36)^*^	−0.003 (1.61)

* *Indicates value that is significantly different from zero*.

A 2 × 6 mixed factorial ANOVA of attitude difference scores considering frame (positive, negative) as the between-subjects factor and replication rate (9, 17, 24, 69, 77, 84%) as the within-subjects factor revealed a significant main effect of frame, *F*_(1, 170)_ = 83.4, *p* < 0.001, $\eta_{p}^{2}$ = 0.329, and a significant main effect of replication rate, *F*_(5, 850)_ = 41.1, *p* < 0.001, $\eta_{p}^{2}$ = 0.195. There was, however, no interaction between frame and replication rate (*p* > 0.39). The lack of a significant interaction between frame and replication rate indicated that the difference in the slopes (reported in Table 2) between the two frame conditions was not statistically significant. Further analysis of the main effect of replication rate revealed a significant linear trend, *F*_(1, 170)_ = 124.8, *p* < 0.001, $\eta_{p}^{2}$ = 0.423. Note that due to the large number of participants in this and subsequent studies, it is likely that a great number of analyses will yield significant differences. Because of this problem, we will not report trend analyses with effect sizes that are less than moderate (i.e., $\eta_{p}^{2}$ < 0.06) as we feel that they have no have any practical meaning. For each replication rate, a single sample *t*-test was conducted to compare the difference score to zero. Here (and elsewhere) we used the Bonferroni correction to maintain the family-wise error rate at 0.05, and the results can be seen in Table 1.

**Table 2:** **Descriptive Statistics for Experiments 1a, 1b, 2, 3, and 4**.

**Experiment**	**Frame**	**Equation**	**X intercept**	**Y intercept**	**Slope**
1a	Positive	y′ = 0.0167x−0.521	31.3	−0.521	0.0167
	Negative	y′ = 0.0205x−1.627	79.4	−1.627	0.0205
1b	Positive	y′ = 0.0129x−0.523	40.4	−0.523	0.0129
	Negative	y′ = 0.0165x−1.673	100	−1.673	0.0166
1a and 1b combined	Positive	y′ = 0.0143x−0.506	35.3	−0.506	0.0143
	Negative	y′ = 0.0179x−1.626	90.7	−1.626	0.0179
2	Positive	y′ = 0.0117x−0.605	51.8	−0.605	0.0117
	Negative	y′ = 0.0143x−1.363	95.5	−1.363	0.0143
3	Positive	y′ = 0.0198x−1.167	59.1	−1.167	0.0198
	Negative	y′ = 0.0099x−0.936	97.5	−0.936	0.0098
4	Positive	y′ = 0.0149x−0.768	51.6	−0.768	0.0149
	Negative	y′ = 0.01436−1.29	90.5	−1.29	0.0143

*For the purpose of calculating these equations all replication rate information was converted to proportions*.

Because the mean attitude change values for the six replication rates reflected a strong linear trend, it was possible to calculate the replication rate associated with no change in attitude by determining the line of best fit through the mean values of the six attitude difference scores for each frame. Some readers may find this calculation useful, as it makes transparent the relative influence of framing and replication rate. This information may be more interesting to science journalists and researchers in the field of science communication than to researchers in the field of judgment and decision making (JDM). When calculating the line of best fit through the mean difference scores for each frame (positive, negative) replication rate was assigned to the x axis and attitude difference to the y axis, as depicted in Figure 1. The resulting equations for the lines of best fit, the x intercept, y intercept and slope values for Experiment 1a (as well as Experiments 1b, 2, 3, and 4) are presented in Table 2. The x intercept is the point at which knowledge of the replication rate has no effect on attitude. Values on the x axis above this level are associated with increasingly favorable attitudes toward science claims, while values below this level are associated with increasingly negative attitudes toward science claims. The slope represents the degree to which participants are sensitive to replication rate information. A slope of 0 would obtain if participants did not revise their attitudes about a science claim at all in the face of information regarding the percentage of studies that had successfully replicated the finding upon which the claim was based. While the slopes calculated for each of the two frames in Experiment 1a are different, the difference is not statistically significant, as indicated by the non-significant interaction between frame and replication rate. The impact of the framing of the information (positive, negative) can be described in two ways. The difference scores in the positive condition were more positive than those in the negative condition (*n* = 96, *M* = 0.256 and *n* = 76, *M* = −0.669, respectively). Described in this way, framing the information in a negative fashion leads (on average) to a difference score 0.925 lower than that expected in the positive condition. The other way of describing the impact of the frame is to compare the x intercept values for the two frame conditions. The x intercept for the positive frame condition is 31.3 while the x intercept for the negative condition is 79.4. This means that when replication rate information is framed in a positive manner, replication rates greater than 31.3% will lead to an increase in favorable attitude toward the claim upon which the research was based. In contrast, when the information is framed negatively, replication rates would have to exceed 79.4% (i.e., 21% or less failed to replicate) before individuals would adopt a more favorable attitude toward the claim.

Experiment 1a confirmed several of our hypotheses. First, the experiment revealed that non-scientists (i.e., students) make use of replication rate information in developing attitudes about science claims. The results also indicate that the relationship between attitude difference and replication rate is roughly linear (H1). That is, attitudes toward a claim increase as a simple linear function of the percentage of attempted replications that were successful. As measured by partial eta-squared, the effect size for frame was even stronger than the effect size for replication rate, indicating that the framing of the information has a very strong impact on attitude (H2).

## Experiment 1b

The six replication rate values employed in Experiment 1a were not evenly spaced and therefore resulted in a number of large gaps. In order to provide a clearer picture of participant response to replication rate information, in Experiment 1b we repeated Experiment 1a using new replication rate values. The values in Experiment 1b were chosen for their ability to “close up” the gaps in the values used in Experiment 1a. As in Experiment 1a percentages that could represent simple ratios (e.g., 25 and 50%) were avoided so that the replication rate would imply that a large number of replications were attempted.

### Methods

#### Participants

Two hundred and thirteen undergraduate students enrolled in psychology courses volunteered to participate. Data from 17 participants were excluded because of failure to answer all the items in the questionnaire and failure to follow instructions. One hundred and sixty of the remaining 196 participants were female. The age range for the sample was 18–48 and the average age was 22.2. Thirty participants were enrolled at a private liberal arts college, 109 were enrolled at a private university, 20 were enrolled at a state college and 37 were enrolled at a community college. All participants were given extra-credit in their psychology courses as compensation.

#### Stimuli and procedure

The stimuli and procedure of Experiment 1b was identical to Experiment 1a with the exception that a different set of replication rate/replication failure rate values were employed. The eight questionnaires presenting replication rate information in a positive frame used six different percentages (4, 32, 42, 54, 92, and 97%) specifying the number of studies that successfully replicated a finding. The eight questionnaires presenting replication rate information in a negative frame employed the corresponding percentages (96, 68, 58, 46, 8, and 3%) specifying the number of studies that failed to replicate a particular finding.

### Results and discussion

Descriptive statistics for Experiment 1b are presented in Table 1 and the pattern of data for Experiment 1b is also presented graphically in Figure 1. A 2 × 6 mixed factorial ANOVA of attitude difference scores considering frame (positive, negative) as the between-subjects factor and replication rate (4, 32, 42, 54, 92, 97%) as the within-subjects factor revealed a significant main effect of frame, *F*_(1, 194)_ = 91.7, *p* < 0.001, $\eta_{p}^{2}$ = 0.321, and a significant main effect of replication rate, *F*_(5, 970)_ = 35, *p* < 0.001, $\eta_{p}^{2}$ = 0.153. There was, however, no interaction between frame and replication rate (*p* > 0.3). The lack of a significant interaction between frame and replication rate indicated that the difference in the slopes for each of the two conditions was not statistically significant. Further analysis of the main effect of replication rate indicated a significant linear trend, *F*_(1, 194)_ = 97.5, *p* < 0.001, $\eta_{p}^{2}$ = 0.334.

## Experiment 2

Experiments 1a and 1b both confirmed the predictions that attitude in relationship to science claims will be linearly related to replication rate (H1) and that the framing of this information will have a big impact (H2). Though the two experiments employed a non-overlapping set of replication rate values, the pattern of results in the two parts of Experiment 1 were very similar to one another. A common criticism of psychological research is that the samples employed in many of the studies are not representative of the population. In regards to Experiments 1a and 1b, this is a valid criticism: the impact of replication rate information and framing on the general population is more interesting than its impact on college students enrolled in introductory psychology courses. In order to address this issue, Experiment 2 was a near-replication of Experiments 1a and 1b using a diverse sample of non-institutionalized U.S. adults above the age of 24. We gathered additional demographic information from participants in Experiment 2 in order to determine if replication rate and framing effects varied as a function of age, education level, income, or self-reported scientific knowledge.

Though many of the participants in Experiment 2 had no college experience, it was predicted that participants in Experiment 2 would, in general, respond to replication rate information in much the same way as the undergraduate college students sampled in Experiments 1a and 1b. It may be that the term “replication” is not well understood by participants with less education or participants with a lower self-reported scientific knowledge. If this is so, then the replication rate effect might be smaller for these participants. However, we are not aware of any studies that show that framing effects are enhanced or attenuated among college students compared to other segments of the population, so we predict that the strong framing effect found in Experiment 1 would be demonstrated in Experiment 2.

### Methods

#### Participants

Two-hundred and twenty-four participants were recruited through an opt-in internet panel managed by a survey research firm (Marketing Systems Group). Data from 22 participants were excluded because the participants completed the survey in <4 min. Data from 14 additional respondents was excluded for failure to answer all the non-demographic items or failure to follow instructions. The remaining participants comprised 188 non-institutionalized adults living in the U.S. The age of respondents ranged from 25 to 83 with a mean of 48.8 and median of 47. Thirty-nine states were represented in the sample and 78 of the respondents were female. Seventy-nine percent of the respondents identified themselves as non-Hispanic white, while 10.1, 8.5, 1.6, and 0.5% identified themselves as black, Hispanic, Asian, and Native American respectively. Additionally, 47.3% of respondents had earned at least one college degree, and 49.2% of the respondents were from households with an annual income below $50 k per year. All participants were paid $2.75 for their participation.

#### Stimuli and procedure

Care was taken to make sure that the on-line questionnaires were as similar as possible to the earlier paper and pencil questionnaires. The stimuli and procedure of Experiment 2 were identical to Experiment 1a with three exceptions. The 16 questionnaire variants created for Experiment 2 contained a more extensive set of demographic questions. Additionally, the questionnaires in Experiment 2 were administered via the internet using Opinio software. In Experiment 2, the questionnaires presenting replication rate information in a positive frame employed six different percentages (4, 17, 42, 69, 84, 97%) specifying the number of studies that successfully replicated a finding. The six percentages were selected from among the set of 12% used in Experiments 1a and 1b and were selected with the goal of choosing values that were evenly spaced. The eight questionnaires presenting replication rate information in a negative frame employed the corresponding percentages (96, 83, 58, 31, 16, 3%) specifying the number of studies that failed to replicate a particular finding.

Several demographic questions followed the presentation of the science claims and responses to these questions were used to categorize participants. In terms of the variable of age, participants were grouped into either the young category (age 25–47, *n* = 93) or old category (age 48+, *n* = 94). In terms of the variable education, participants were categorized on the basis of whether they had (*n* = 89) or had not (*n* = 99) earned a bachelor's degree. In terms of the variable knowledge, participants were categorized based on how much knowledge they felt they had in regards to science. The low knowledge group (*n* = 120) included all participants that indicated that they were either not very knowledgeable or somewhat knowledgeable about science and the high knowledge group (*n* = 68) included all participants who indicated that they were either moderately knowledgeable or very knowledgeable about science. In terms of the variable income, participants were grouped into either the low income category (<$50 k/yr, *n* = 92) or the high income category ($50 k/yr or more, *n* = 95).

### Results and discussion

**Figure 2:**
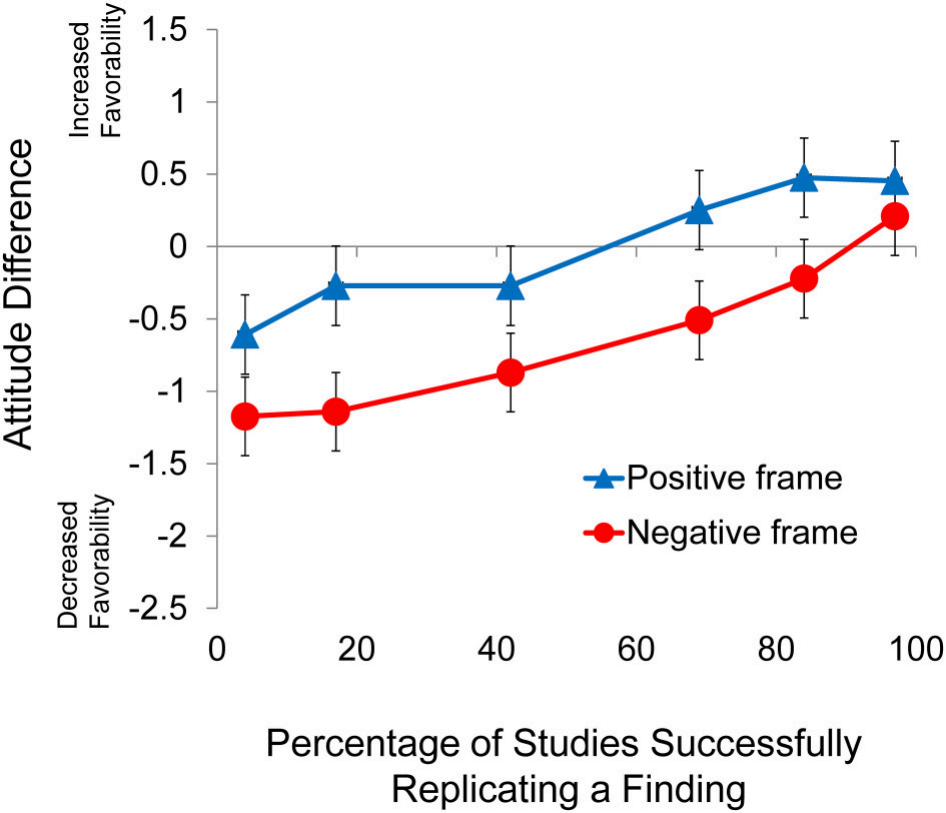
**Difference score as a function of frame for Experiment 2**. Error bars indicate 95% confidence intervals.

As with Experiments 1a, 1b, 3, and 4, preliminary analyses involving age and gender were conducted for Experiment 1. In addition to those analyses, we also conducted analyses of knowledge, income, and education. The preliminary analyses for Experiment 2 revealed no significant effects involving any of these five subject variables (*p* > 0.21 in all cases). Therefore, we report analyses of the data in aggregate form. Descriptive statistics for Experiment 2 are presented in Table 3 and the pattern of data for Experiment 2 is presented graphically in Figure 2. A 2 × 6 mixed factorial ANOVA considering frame (positive, negative) as the between-subjects factor and replication rate (4, 17, 42, 69, 84, 97%) as the within-subjects factor revealed a significant main effect of frame, *F*_(1, 186)_ = 36.2, *p* < 0.001, $\eta_{p}^{2}$ = 0.163, and a significant main effect of replication rate, *F*_(5, 970)_ = 29.1, *p* < 0.001, $\eta_{p}^{2}$ = 0.135. There was, however, no interaction between frame and replication rate (*p* > 0.2). The lack of a significant interaction between frame and replication rate indicated that the difference in the slopes for each of the two conditions was not statistically significant. Further analysis of the main effect of replication rate revealed a significant linear trend, *F*_(1, 186)_ = 85.4, *p* < 0.001, $\eta_{p}^{2}$ = 0.315.

**Table 3:** **Mean and standard deviation of difference scores for Experiment 2**.

**Replication rate (%)**	**Positive frame *M(SD)***	**Negative frame *M(SD)***
4	−0.608 (1.42)^*^	−1.174 (1.50)^*^
17	−0.271 (1.27)	−1.141 (1.30)^*^
42	−0.271 (1.29)	−0.871 (1.17)^*^
69	0.252 (1.28)	−0.509 (1.96)^*^
84	0.476 (1.36)^*^	−0.222 (1.38)
97	0.454 (1.55)	0.210 (1.30)

* *Indicates value that is significantly different from zero*.

Even though the samples included in Experiments 1 and 2 were very different, the results of both studies paint a very consistent picture: replication rate and framing both have a strong impact on attitude. Note also that a different set of six replication rates were employed in Experiments 1a, 1b, and 2, indicating that the pattern of results is not due to an artifact related to a particular set of replication rates chosen.

## Experiment 3

Some have suggested that humans are better able to process information when it is presented in the format of natural frequencies (Gigerenzer and Hoffrage, 1995; Cosmides and Tooby, 1996). To address this possibility, in Experiment 3 the replication rate information was presented in the form of natural frequencies (e.g., “4 of the 10 studies that attempted to replicate the findings succeeded in doing so”). Throughout Experiment 3 the total number of attempted replications was always 10. Note that the replication rates employed in the earlier studies (e.g., 97%) implied that far more than 10 research teams attempted to replicate each finding. In general, the natural frequencies reported in Experiment 3 used Arabic numerals (e.g., 4 of 10). As we constructed the stimuli, however, we noticed that the phrases “0 out of 10” and “10 out of 10” could be awkward and unnatural. We therefore replaced Arabic numerals with words in some of the 0 of 10 and 10 of 10 versions of the stimuli.

One goal of Experiment 3 was to determine if changing the format in which the replication rate is presented would have any impact on attitude (H3). Due to the fact that natural frequencies are easier to work with and result in fewer reasoning “errors,” it was our hypothesis that the framing effects seen in Experiments 1 and 2 would be attenuated in Experiment 3. Let us assume that the more dubious a finding is, the greater the number of research teams will attempt to replicate the effect. Since the studies described in Experiment 3 only resulted in 10 research teams attempting replication while the studies reported in Experiments 1 and 2 involved much larger numbers of teams attempting replication, our non-scientist participants may have more confidence in the findings reported in Experiment 3.

We predicted that there will be no difference in attitude as a function of the form in which the natural frequency data is presented (Arabic numerals vs. words) (H4). If this is the case, then the data from the 0/none and 10/all conditions can be combined and analyzed along with the other four conditions. If the form in which the numeric data is presented does impact attitude, however, then those conditions must be analyzed separately.

### Methods

#### Participants

Two hundred undergraduate students enrolled in psychology courses volunteered to participate. Data from 14 participants were excluded because of failure to answer all the items in the questionnaire and/or failure to follow instructions. One hundred and twenty-seven of the remaining 186 participants were female. The age range for the sample was 18–55 and the average age was 22.4. Fifty-seven participants were students enrolled at a private liberal arts college, 124 were enrolled at a community college, and 5 participants did not indicate the institution they were attending. All participants were given extra-credit in their psychology courses as compensation.

#### Stimuli and procedure

The stimuli and procedure of Experiments 3 were identical to Experiment 1 with several exceptions. All participants in Experiment 3 completed an on-line questionnaire generated by Opinio. Additionally, the replication rate information was presented in the form of natural frequencies, rather than percentages. In all cases, the additional information indicated that 10 studies had been conducted in an attempt to replicate a key study upon which the initial claim was based. Questionnaires presenting replication rate information in a positive frame employed six different ratios (0 of 10, 2 of 10, 4 of 10, 6 of 10, 8 of 10, and 10 of 10) specifying the number of studies that successfully replicated a finding. Questionnaires presenting replication rate information in a negative frame employed six different ratios (10 of 10, 8 of 10, 6 of 10, 4 of 10, 2 of 10, and 0 of 10) specifying the number of studies that failed to replicate a finding. The wording in some items was slightly altered in order to accommodate the change from percentages to natural frequencies (see Tables S4, S5). For some of the conditions (i.e., 2 of 10, 4 of 10, 6 of 10, and 8 of 10) natural frequencies were always presented with Arabic numerals. For the 0 of 10 and 10 of 10 conditions, however, Arabic numerals (e.g., 0 of 10) were used for some items while words (e.g., none of the 10) were used for other items.

### Results and discussion

#### Analysis of conditions only using natural frequencies

**Figure 3:**
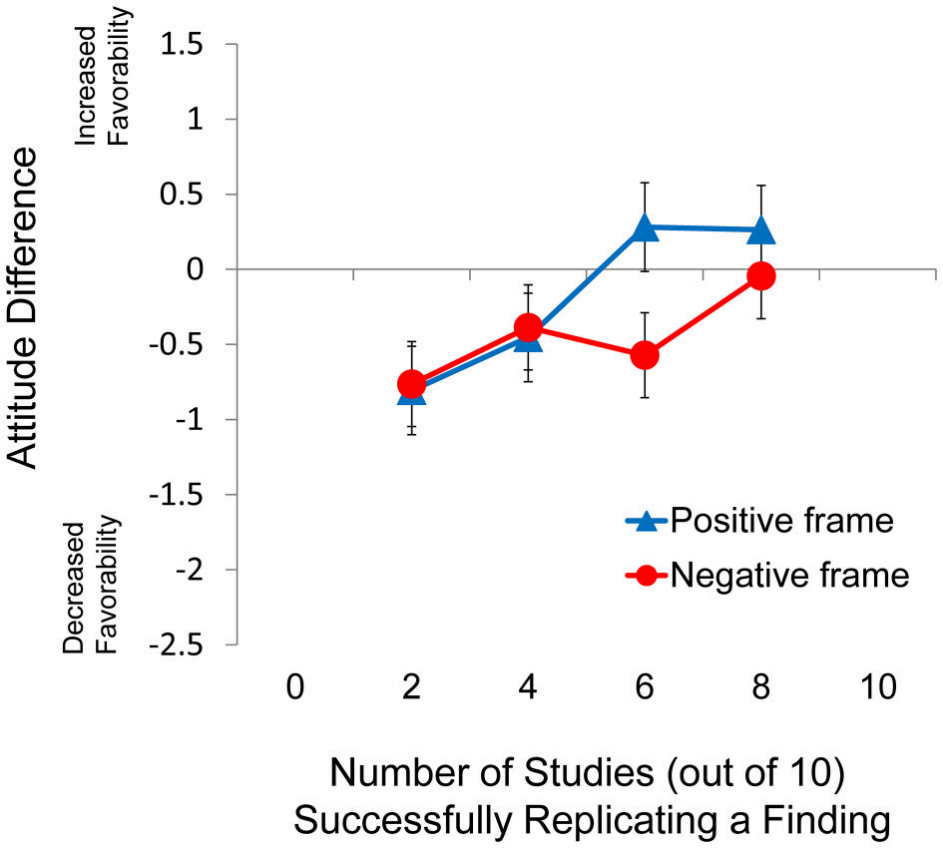
**Difference score as a function of frame for Experiment 3**. Error bars indicate 95% confidence intervals.

Descriptive statistics for Experiment 3 are presented in Table 4 and the pattern of data for Experiment 3 is also presented graphically in Figure 3. Four levels of replication rate (2, 4, 6, and 8 out of 10) were always presented with Arabic numerals. In the 10 of 10 and 0 of 10 conditions, however, half of the items were presented with Arabic numerals (10, 0) while the rest were presented with the equivalent words (all, none). Therefore, the conditions that included two different kinds of stimuli to indicate natural frequencies were analyzed separately using independent samples *t*-tests and the descriptive statistics for these conditions are summarized in Table 5.

**Table 4:** **Mean and standard deviation of difference scores for Experiment 3**.

**Replication rate**	**Positive frame *M(SD)***	**Negative frame *M(SD)***
2 of 10	−0.807 (1.42)^*^	−0.764 (1.3)^*^
4 of 10	−0.453 (1.26)^*^	−0.387 (1.27)^*^
6 of 10	0.281 (1.28)	−0.572 (1.27)^*^
8 of 10	0.264 (1.59)	−0.045 (1.39)

* *Indicates value that is significantly different from zero*.

**Table 5:** **Descriptive statistics for the 0 of 10 and 10 of 10 replication rate conditions of Experiment 3**.

**Frame**	**Replication rate**	**Stimuli**	***n***	***M(SD)***	**Distribution**
Positive	0 of 10 succeed^*^	0	45	−1.642 (1.59)	Unimodal
		None	44	−1.017 (1.29)	Bimodal with peaks at 0.5 and −1.5
	10 of 10 succeed	10	56	0.667 (1.45)	Unimodal
		All	33	0.821 (1.67)	Unimodal
Negative	0 of 10 succeed^*^	10	61	−1.754 (1.21)	Bimodal with peaks at 2 and −2
		All	36	−1.065 (1.53)	Flat from −0.5 to 2.5
	10 of 10 Succeed^*^	0	60	−0.623 (1.76)	Unimodal
		None	37	0.200 (1.73)	Unimodal

* *Indicates significant difference at 0.05 level*.

To analyze these four levels of replication rate, a 2 × 4 mixed factorial ANOVA considering frame (positive, negative) as the between-subjects factor and replication rate (2 of 10, 4 of 10, 6 of 10, 8 of 10) as the within-subjects factor revealed a significant effect of frame, *F*_(1, 184)_ = 4.76, *p* = 0.03, $\eta_{p}^{2}$ = 0.025, and a significant main effect of replication rate, *F*_(3, 552)_ = 17.9, *p* < 0.001, $\eta_{p}^{2}$ = 0.09. There was also an interaction between frame and replication rate, *F*_(3, 552)_ = 5.61, *p* = 0.001, $\eta_{p}^{2}$ = 0.03. An examination of the means revealed that the frame effect was most pronounced when the replication rate was 6 out of 10. However, the effect size associated with this interaction was weak and therefore the interaction may not have any practical meaning. Further analysis of the main effect of replication rate revealed a significant linear trend, *F*_(1, 184)_ = 40.2, *p* < 0.001, $\eta_{p}^{2}$ = 0.179.

Though the ANOVA revealed both a significant main effect of frame and a significant interaction between frame and replication rate, the related effect sizes were much smaller than the effect size of the framing effect seen in Experiments 1 and 2. Due to the large n of the study and the relatively small effect sizes, we consider the statistically significant results to have little practical significance.

#### Analysis of conditions using natural frequencies and arabic numerals

As noted above, in two levels of replication rate (0 of 10; 10 of 10) some questionnaire items included Arabic numerals (10, 0) while others included the equivalent words (all, none). Therefore, these conditions were analyzed separately using independent samples *t*-tests and the descriptive statistics for these conditions are summarized in Table 5. In three of the four comparisons (positive frame, 0 of 10, 10 of 10; negative frame, 10 of 10) the Arabic numeral version was significantly different form the equivalent information presented with a word (*p* < 0.045 in all cases). The results of the independent samples *t*-tests justify our omission of the 10 of 10 and 0 of 10 conditions from the earlier analysis of variance.

Perhaps more interesting than the results of the independent samples *t*-tests, however, were the distributions of difference scores in the 0 of 10 replication rate conditions (see the “distribution” column in Table 5). Note that two out of four of these conditions were characterized by bimodal distributions. Before going on, it should be noted that none of the 2, 4, 6, or 8 out of 10 conditions of Experiment 3 were characterized by bimodal distributions, nor were any of the condition in Experiments 1a, 1b, or 2. The statements associated with bimodal distributions included “none of the 10 succeeded in replicating” (positive frame) and “10/all of 10 failed to replicate” (negative frame). Note that the only condition containing a double negative “0/none of 10 failed to replicate” (negative frame) was characterized by a unimodal distribution. The pattern of unimodal and bimodal distributions further justifies our omission of the 10 of 10 and 0 of 10 conditions from the earlier ANOVA.

In Experiment 3, as in Experiments 1 and 2, there was an effect of replication rate, characterized by a strong linear trend, (H1) and an effect of frame (H2). Unlike the previous experiments, however, the effect of frame in Experiment 3 was much weaker and therefore might have little practical relevance. The smaller magnitude of the framing effect in Experiment 3 appears to lend some support our prediction that natural frequencies would be associated with an attenuated framing effect (H3). The significant main effect of replication rate ruled out the possibility that participant attitude was only influenced by set size. However, we cannot determine if participants were influenced by the ratio between the subset and the entire set or if they were influenced only by the size of the subset. In either case, one would expect to see a strong linear trend of replication rate.

## Experiment 4

In Experiment 3, participants were always informed that 10 research teams had attempted to replicate each finding. The results of Experiment 3 differed from Experiments 1 in 2 in that they did not reveal a strong framing effect. The lack of framing effect in Experiment 3 may be an artifact due to the specific natural frequencies employed. In Experiment 4, participants were informed that 5, not 10, research teams attempted to replicate each finding. Experiment 4 has the potential to confirm the attenuated framing effect (H3) seen in Experiment 3. Additionally, Experiment 4 may confirm the unexpected pattern of results found in the 0 of 10 and 10 of 10 conditions of Experiment 3 (H4).

### Methods

#### Participants

One hundred and ninety-six undergraduate students enrolled in psychology courses volunteered to participate. Data from 20 participants were excluded because of failure to answer all the items in the questionnaire and/or failure to follow instructions. One hundred and twenty-seven of the remaining 177 participants were female. The age range for the sample was 18–55 and the average age was 26.3. Five participants were students enrolled at a private liberal arts college, 166 were enrolled at a community college, and 5 participants did not indicate the institution they were attending. All participants were given extra credit in their psychology courses as compensation.

#### Stimuli and procedure

The stimuli and procedure of Experiments 4 was identical to Experiment 3 with several exceptions. In all cases, the additional information indicated that five studies had attempted to replicate a key study upon which the initial claim was based. Questionnaires presenting replication rate information in a positive frame employed six different ratios (0 of 5, 1 of 5, 2 of 5, 3 of 5, 4 of 5, and 5 of 5) specifying the number of studies that successfully replicated a finding. Questionnaires presenting replication rate information in a negative frame employed six different ratios (5 of 5, 4 of 5, 3 of 5, 2 of 5, 1 of 5, and 0 of 5) specifying the number of studies that failed to replicate a finding. As with Experiment 3, words were substituted for Arabic numerals for some items (H4). The Experiment 4 substitutions occurred in the same places as the substitutions of Experiment 3 (see Tables S4, S5).

### Results and discussion

#### Analysis of conditions only using natural frequencies

**Figure 4:**
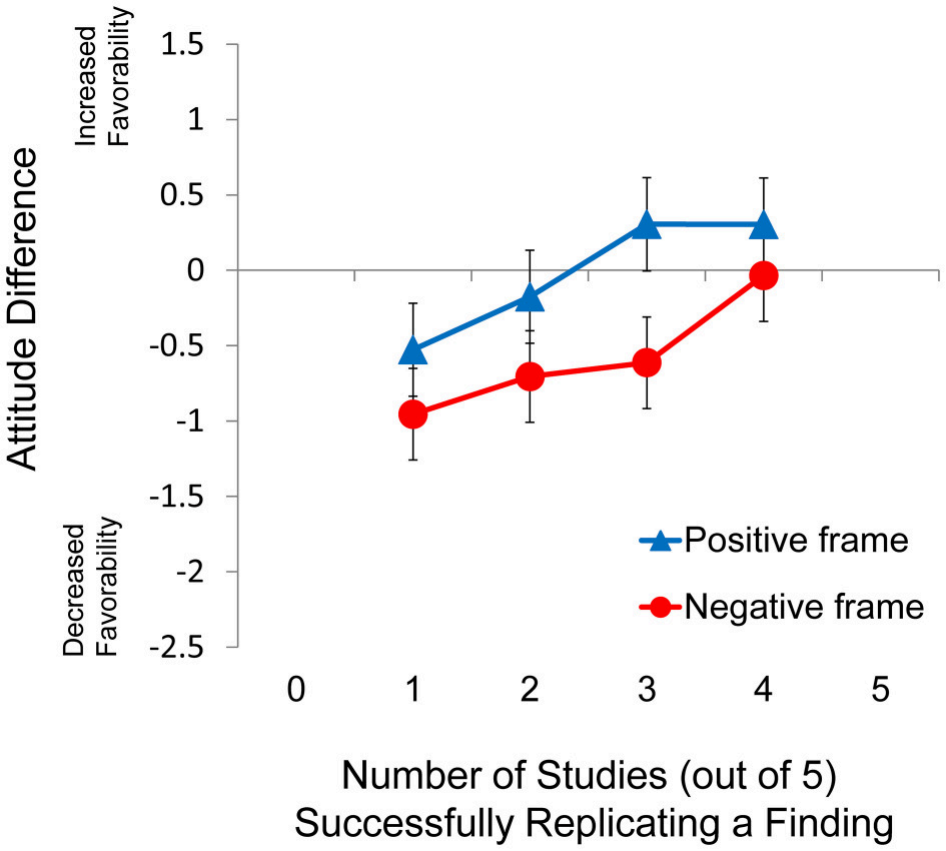
**Difference score as a function of frame for Experiment 4**. Error bars indicate 95% confidence intervals.

Descriptive statistics for the 1 of 5, 2 of 5, 3 of 5, and 4 of 5 conditions, in which natural frequencies were always presented with Arabic numerals, are presented in Table 6 and the pattern of data for these conditions is also presented graphically in Figure 4. A 2 × 4 mixed factorial ANOVA considering frame (positive, negative) as the between-subjects factor and replication rate (1 of 5, 2 of 5, 3 of 5, 4 of 5) as the within-subjects factor revealed a significant main effect of frame, *F*_(1, 175)_ = 19, *p* < 0.001, $\eta_{p}^{2}$ = 0.098, and a significant main effect of replication rate, *F*_(3, 525)_ = 15.5, *p* < 0.001, $\eta_{p}^{2}$ = 0.081. There was no interaction between frame and replication rate, *F*_(3, 525)_ = 1.78, *p* > 0.15, $\eta_{p}^{2}$ = 0.01. Further analysis of the main effect of replication rate indicated a significant linear trend, *F*_(1, 175)_ = 40.1, *p* < 0.001, $\eta_{p}^{2}$ = 0.187.

**Table 6:** **Mean and standard deviation of difference scores for Experiment 4**.

**Replication rate**	**Positive frame *M(SD)***	**Negative frame *M(SD)***
1 of 5	−0.527 (1.29)^*^	−0.955 (1.43)^*^
2 of 5	−0.176 (1.33)	−0.705 (1.26)^*^
3 of 5	0.305 (1.31)	−0.613 (1.40)^*^
4 of 5	0.304 (1.50)	−0.035 (1.54)

* *Indicates value that is significantly different from zero*.

#### Analysis of conditions using natural frequencies and arabic numerals

In the 5 of 5 and 0 of 5 conditions some items included Arabic numerals (0, 5) while others included the equivalent words (none, all). These conditions were analyzed separately using independent samples *t*-tests and the descriptive statistics are summarized in Table 7. No significant differences were found between the Arabic numeral and words in any of the conditions (*p* > 0.09 in all cases). In contrast to Experiment 3, the results of the independent samples *t*-tests do not appear to justify our omission of the 5 of 5 and 0 of 5 conditions from the earlier analysis of variance.

**Table 7:** **Descriptive statistics for the 0 of 5 and 5 of 5 replication rate conditions of Experiment 4**.

**Frame**	**Replication rate**	**Stimuli**	***n***	***M(SD)***	**Distribution**
Positive	0 of 5 succeed	0	47	−1.202 (1.42)	Bimodal with peaks at 0 and −2.5
		None	40	−1.196 (1.44)	Flat from −3 to −0.5
	5 of 5 succeed	5	56	0.800 (1.58)	Unimodal
		All	31	0.189 (1.64)	Unimodal
Negative	0 of 5 succeed	5	60	−1.596(1.4)	Bimodal with peaks at −2.5 and 2.5
		All	30	−1.095(1.75)	Bimodal with peaks at −2.5 and 0.5
	5 of 5 Succeed	0	57	−0.701(1.65)	Unimodal
		None	33	−0.152(2.12)	Unimodal

The pattern of distributions for the four conditions do, however, justify the omission of the 0 of 5 and 5 of 5 conditions from the 2 × 4 mixed factorial ANOVA of the Experiment 4 difference scores. For the replication rate of 0 of 5 there were four conditions (positive Arabic, negative Arabic, positive word, negative word) and three of these conditions were characterized by bimodal distributions (the fourth was characterized by a flat distribution). This is in contrast to the 1 of 5, 2 of 5, 3 of 5, 4 of 5, and 5 of 5 conditions of Experiment 4 that were not characterized by bimodal or flat distributions. In a pattern similar to that found in Experiment 3, the statements associated with bimodal distributions included “0/none of the 5 succeeded in replicating” (positive frame) and “5/all of 5 failed to replicate” (negative frame). As in Experiment 3, the only condition containing a double negative “0/none of 5 failed to replicate” (negative frame) was characterized by a unimodal distribution. These results indicate that the findings of Experiment 3 were not a fluke: the manner in which natural frequencies are presented (Arabic numerals vs. words) does make a difference to participants.

#### Experiments 3 and 4 compared to experiments 1 and 2

The results of Experiment 3 indicate that a much smaller framing effect obtained when natural frequencies were employed. A statistically significant and practically meaningful framing effect was found in Experiment 4; however, this effect was smaller than that found in either Experiments 1 or 2. Though the effect size of framing was much larger when the replication rate information was presented in the form of percentages, it is clear that the use of natural frequencies does not actually eliminate the effect.

## Experiment 5

Experiments 1 through 4 provided a great deal of information in regards to how attitude is affected by replication rate information, however, there are two limitations of Experiments 1 through 4. These experiments do not demonstrate that the effects would also obtain in tasks employing choice as the dependent measure, nor do they rule out the possibility that the effects might be due to participants being presented with different percentages within the same questionnaire. Regarding the first point: although Kahneman et al. (1999) have argued that the willingness to pay (a decision-based dependent measure) is appropriately viewed as equivalent to an expression of attitude, we cannot be certain that the findings of Experiments 1 through 4 will generalize to a task that relies on choice as a dependent measure. The main variables employed in the first four experiments [replication rate, frame (positive, negative), and numeric format (percentage, natural frequency)] were included in Experiment 5. Participants in Experiment 5 were not asked for their attitude in regards to a science claim, however. Instead they were presented with evidence for and against a pair of prescription drugs that could be used to treat the same symptoms. After reading about the two drugs, participants indicated which of the two drugs they would choose in order to treat these symptoms.

It is our hypothesis that participants will be more likely to choose a particular drug if positive findings in regards to this are associated with a large replication rate (H1) and if the replication rate is framed in a positive manner (H2). We also predict that the framing effect will be larger in the percentage conditions as compared to the natural frequency conditions (H3).

### Methods

#### Participants

Participants were 458 students enrolled at a community college and 204 college students enrolled at a state university. All students received extra-credit in their psychology courses for their participation. Four hundred and sixty-four participants were female; the age range for the sample was 18–60 and the average age was 24.1.

#### Stimuli and procedure

All community college participants completed an on-line questionnaire created using Opinio software. Participants at the state university completed the same questionnaire on paper. All participants read the following scenario about two drugs:

“Assume that you have just visited your physician where you learned that you suffer from very high blood pressure. Your physician is willing to write out a prescription for one of two drugs to treat high blood pressure: Ansulfazor or Trocamazor.

You know this about Ansulfazor: An experiment conducted by Dr. Howel revealed that Ansulfazor was slightly more effective than Trocamazor, but Ansuulfazor had somewhat more side-effects than Trocamazor.

You know this about Trocamazor: An experiment conducted by Dr. Simonson indicated that Trocamazor was much more effective than Ansulfazor.”

A final sentence always followed the information presented above. There were 12 versions of this final sentence and they differed in terms of replication rate (8%, 43%, 81%, 92%, 4 of 10, 2 of 5) and frame (positive, negative). Each participant was presented with only 1 of the 12 variations, as Experiment 5 was a completely between-subjects design. The sentence included in the 43% replication rate condition surveys was, “Additionally, 43% of the researchers who tried to replicate Dr. Simonson's findings succeeded in doing so.” The wording for the other 11 conditions can be found in Table S6.

Participants were asked to imagine that they had to choose one of these two drugs and then indicate the one that they would choose (this constituted the sole dependent measure). Once participants indicated their choice (or clicked “next” to move to the next screen) they were presented with a series of demographic questions identical to those in Experiments 3 and 4.

### Results

Descriptive statistics for Experiment 5 are presented in Table 8 and a summary of chi-squared analyses for Experiment 5 are presented in Table 9. Chi-squared tests revealed a significant framing effect for five of the six replication rate conditions (*p* < 0.001 in all five cases). The framing effect was not significant in the 8% replication rate condition, although the data were in the predicted direction. The significant results for the natural frequency conditions indicate that, in tasks in which choice serves as the dependent measure, framing effects are not attenuated when the replication rate information is communicated in terms of natural frequency.

**Table 8:** **Descriptive statistics for Experiment 5: percentage of sample favoring each drug as a function of replication rate and frame**.

**Replication rate (%)**	**Frame**	**Drug choice**	
		**Ansulfazor**	**Trocamazor**
8	Positive	38 (19)	62 (31)
	Negative	46 (22)	54 (26)
43	Positive	20 (11)	80 (45)
	Negative	57 (33)	43 (25)
81	Positive	10 (5)	90 (45)
	Negative	51 (31)	49 (30)
92	Positive	7 (4)	93 (50)
	Negative	58 (30)	42 (22)
2 of 5	Positive	19 (10)	81 (43)
	Negative	54 (34)	46 (29)
4 of 10	Positive	18 (11)	82 (51)
	Negative	64 (35)	36 (20)

*Values in parentheses are cell sample sizes*.

**Table 9:** **Summary of chi-squared analyses for Experiment 5**.

**Comparison**	**Statistic**
8% positive vs. 8% negative	χ^2^ (1, *n* = 97) = 0.62, *p* = 0.43
43% positive vs. 43% negative	χ^2^ (1, *n* = 114) = 16.7, *p* < 0.001
81% positive vs. 81% negative	χ^2^ (1, *n* = 111) = 20.9, *p* < 0.001
92% positive vs. 92% negative	χ^2^ (1, *n* = 105) = 30.7, *p* < 0.001
2 of 5 positive vs. 2 of 5 negative	χ^2^ (1, *n* = 116) = 15.1, *p* < 0.001
4 of 10 positive vs. 4 of 10 negative	χ^2^ (1, *n* = 117) = 25.7, *p* < 0.001
8% positive vs. 43% positive	χ^2^ (1, *n* = 105) = 4.4, *p* = 0.04
8% positive vs. 81% positive	χ^2^ (1, *n* = 99) = 10.8, *p* = 0.001
8% positive vs. 92% positive	χ^2^ (1, *n* = 103) = 14.1, *p* < 0.001
43% positive vs. 81% positive	χ^2^ (1, *n* = 105) = 1.9, *p* = 0.17
43% positive vs. 92% positive	χ^2^ (1, *n* = 109) = 3.5, *p* = 0.06
81% positive vs. 92% positive	χ^2^ (1, *n* = 103) = 0.2, *p* = 0.64
8% negative vs. 43% negative	χ^2^ (1, *n* = 105) = 1.3, *p* = 0.26
8% negative vs. 81% negative	χ^2^ (1, *n* = 108) = 0.27, *p* = 0.67
8% negative vs. 92% negative	χ^2^ (1, *n* = 99) = 1.4, *p* = 0.24
43% negative vs. 81% negative	χ^2^ (1, *n* = 118) = 0.4, *p* = 0.51
43% negative vs. 92% negative	χ^2^ (1, *n* = 109) <0.01, *p* = 0.93
81% negative vs. 92% negative	χ^2^ (1, *n* = 112) = 0.5, *p* = 0.47

A series of chi-squared analyses were conducted to explore the possibility that the choice task used in Experiment 5 would reveal replication rate effects. These analyses were not conducted on the natural frequency conditions of Experiment 5, as the two conditions (4 of 10, 2 of 5) contain the same replication rate information. For the positive frame, percentage replication rate conditions, all replication rates were compared to all the other replication rates using individual chi-squared tests. The data from all comparisons of the positively framed data were in a direction consistent with a replication rate effect, though not all comparisons reached statistical significance. All comparisons were significant at the 0.05 level (*p*-values ranged from <0.001 to 0.04) except for three. The 43 vs. 92% comparison did not quite reach the 0.05 level (*p* = 0.06). Additionally, the 43 vs. 81% and the 81 vs. 92% comparisons were non-significant (*p* = 0.17 and 0.6 respectively). Chi-squared tests were also used to determine if similar replication rate effects were found for the negative frame, percentage replication rate conditions. In contrast to the analyses of the positive frame condition, no significant effects of replication rate were found in the negative frame condition (*p*-values ranged from 0.24 to 0.93), however. It may be that the negative frame wording in the choice task was so confusing that participants simply chose an option at random.

## General discussion

The results of five studies generally supported three of our four hypotheses. Our samples of college students and members of the general public responded to replication rate information in a sensible fashion: the lower the replication rate of a study the less favorable attitudes toward a claim based on that study (H1). There was an even stronger effect of framing, however: participants had more favorable attitudes toward science claims when the replication rate was framed in a positive, rather than a negative, fashion (H2). The framing effect found in the studies employing attitude difference as a dependent measure was replicated in a study in which choice served as the dependent measure. The sensitivity to replication rate was found regardless of whether the replication rate was presented as a percentage or a natural frequency. Based on effect size, x-intercept values, and the mean values of the positive and negative frame conditions, the framing effect appeared to be more pronounced in the percentage experiments than in the two natural frequency experiments (H3). While this suggests the possibility that the framing effect might be attenuated when replication rate information is presented in natural frequency format, we found no support for H3 when choice served as the dependent measure. Finally, results of Experiments 3 and 4 indicated that the format that the natural frequencies were presented in (words vs. numbers) did have an impact on subjects, and this finding was evidence against H4.

The examples employed in the questionnaires involved natural rather than social science claims. We do not know if our hypotheses would have been supported if we had employed social science claims in lieu of natural science claims. Additionally, the results of the current studies cannot be compared to an objective benchmark. We don't know the “right” or “rational” amount that attitude regarding a science claim ought to be altered in the face of information about replication rate. So while the results may speak to such issues as how non-scientists are influenced by framing and replication rate information, the results can't tell us whether-or-not the participants were being influenced in the “correct” fashion.

When replication rate information was presented in natural frequency format (Experiments 3 and 4) both the number of attempted replications and the quantity of the subset of the sample that succeeded/failed to replicate the finding were available. In responding to science claims paired with replication rate information presented in a natural frequency format, participants could use (A) both pieces of information, (B) neither piece of information, (C) the quantity of the subset only, or (D) the number of attempted replications only. The strong effect of replication rate in both experiments ruled out options B and D. Because we could not run statistical analyses comparing Experiments 3 and 4, we cannot rule out option C. However, we feel that the data better support option A; that participant attitude was influenced by the ratio between replications and attempted replications as the most likely factor influencing participant opinion.

The presence of a strong framing effect in all five studies is consistent with the findings of most studies involving what Levin et al. (1998) refer to as attribution frames. The general finding of these studies is that a particular alternative is rated more favorably when described positively than when described negatively. Experiments 1 through 4 extended previous research on this topic by allowing us to compare effect size of framing with effect size of replication rate. We not only have demonstrated that framing is more influential than replication rate, we have shown how much more influential it is. Given our results, one could predict that a U.S. adult who read a New York Times article claiming that 10% of the attempted replications of study X have failed to replicate the finding, would have the same attitude about study X as a similar individual who read a Wall Street Journal article claiming that 45% of the attempted replications of study X have been successful. Studies 1 through 4 indicate that a scientist or science journalist communicating to members of the general public may be able to minimize the impact of framing by communicating replication rate information as natural frequencies rather than percentages.

On the one hand, the results of our attitude experiments suggest that natural frequencies are associated with an attenuated framing effect. On the other hand, there was not any evidence that natural frequencies attenuate the framing effect in the choice tasks. Even in the tasks using attitude as a dependent measure, there was still a significant framing effect in the natural frequencies conditions and the difference in the magnitude of the framing effect between the percentage and the natural frequencies conditions was not large.

Although there is a long and rich tradition exploring the manner in which individuals use and respond to qualitative probability expressions and quantitative probability values (Toogood, 1980; Beyth-Marom, 1982; Nakao and Axelrod, 1983; Budescu and Wallsten, 1985; Mosteller and Youtz, 1990), the literature is silent in regards to the impact of using Arabic numerals or equivalent words in natural frequency expressions (e.g., 5 of 5 vs. all of 5). The finding in Experiment 3 that the impact of some natural frequency expressions was influenced by the manner in which the quantity of the subcategory was described (Arabic numeral vs. word) was unexpected and difficult to integrate with the literature on natural frequencies. The effect was not replicated in Experiment 4 (although the trends were in the same direction) so it is difficult to interpret the findings.

Even though framing effects are usually established in studies employing choices or decisions as dependent measures, we relied primarily on tasks involving attitude as the dependent measure. In the one experiment in which choice served as the dependent measure (Experiment 5), we replicated the framing effect and partially replicated the replication rate effect. Both the attitude and choice tasks indicated that framing is more important than replication rate. Kahneman et al. (1999) developed an argument that patterns of results found in tasks employing the dependent measures of choice and contingent valuation (CVM) ought to be found in tasks using attitude as a dependent measure. Kahneman et al. (1999) claimed that choice is a special case of comparative valuation and argue that attitude and CVM responses are highly correlated and that the same things that affect dollar valuations (frames, anchors, etc.) tend to also affect attitudes as well (see also Kahneman and Ritov, 1994; Kahneman et al., 1998; Payne et al., 1999). The fact that we partially replicated the results of experiments 1–4 with a study employing choice as the dependent measure is consistent with the perspective of Kahneman et al. (1999).

The current studies found that agreement with a claim increases with the percentage of researchers who replicate (and thus agree with) the claim. The results of the current studies have so far been discussed terms of JDM and science communication. However, social learning researchers have found empirical evidence of learning through strategies that include “copy the majority” (Boyd and Richerson, 1985; Pike and Laland, 2010) and “copy the behavior with the highest absolute number of demonstrators” (Bond, 2005). To the degree that attitude change is related to behavioral change, the replication rate effect in the present studies might suggest a way to form a bridge between JDM and social learning approaches. The results of the current studies are consistent with past studies that have explored attribute framing, but the current studies are unique in that they demonstrate an effect of framing upon interpretations of a given replication rate and do so using both attitude and choice as dependent measures. There are important practical implications of the rather large framing effect shown in these studies. Scientists, politicians, policy makers, journalists, corporations, and think tanks use scientific findings in order to influence public opinion about a wide range of critical issues. The current studies indicate that non-scientists are strongly influenced by knowledge of replication, and the studies also illustrate that the framing of the replication rate of a given scientific finding is an important tool that communicators can exploit. In fact, the current results suggest that public opinion in regards to an issue such as global warming may be influenced just as much by the empirical findings, as by the manner in which the findings are framed.

## Author contributions

All authors listed, have made substantial, direct and intellectual contribution to the work, and approved it for publication.

### Conflict of interest statement

The authors declare that the research was conducted in the absence of any commercial or financial relationships that could be construed as a potential conflict of interest. The reviewer MF and the handling Editor declared their shared affiliation, and the handling Editor states that the process nevertheless met the standards of a fair and objective review.
