# Supplementary material for: Replication Rate, Framing, and Format Affect Attitudes and Decisions about Science Claims
Source: Front Psychol. 2016 Nov 22;7:1826. doi: 10.3389/fpsyg.2016.01826 (PMC5118435; doi:10.3389/fpsyg.2016.01826)
Supplement: Supplementary file 1 [file DataSheet1.pdf]

Table S1. Initial Claims for Experiments 1, 2, 3 and 4.

1	According to a research team at U.C.L.A., infants that consumed formula that was supplemented with lutein had higher I.Q. scores at age 10 compared to infants that consumed regular formula. Therefore, the nutritional supplement lutein, when given to infants enhances brain development.
2	According to two prominent psychiatrists, a new drug, Prevadarin, effectively treats obsessive compulsive disorder.
3	A team of African biologists found that creating a series of medium sized animal preserves throughout a rainforest drastically decreases the poaching of apes and monkeys by hunters.
4	A team of astronomers at the Chandra X-ray Observatory found that the sun emits 30 times more X-rays than most other stars of the same size and age.
5	A group of biologists at Washington State University recently compared the DNA of jaguars with that of other great cats. They found the highest degree of similarity between jaguars and lynxes and concluded that the lynx is the closest relative of the jaguar.
6	A team of researchers at City College of New York concluded from the rock samples they analyzed that 34,000 years ago Manhattan was not an island but was part of the mainland.
7	Magnesium diboride becomes a superconductor at -126 degrees Celsius.
8	Direct electrical stimulation of the splanchnic nerve is effective at treating cases of severe depression.
9	Clusters of prions in the brain cause Lou Gehrig's disease.

10 The decline of the population of fresh water perch in Lake Erie is due to competition from snipe eels, a non-native species that was introduced to the Lake in the 1990s.

11 Laparoscopic surgery results in high levels of post operative infection when the technique is used to remove a person’s appendix.

12 A new drug, Amgonalen, effectively treats schizoid personality disorder.

Table S2. Additional Information for Experiment 1a, Positive Frame.

1 A number of research teams have looked for a connection between lutein and brain development and [9%, 17%, 24%, 69%, 77%, 84%] have found a benefit for lutein.

2 Researchers at a number of universities tested Prevadarin for its effect on obsessive compulsive disorder and [9%, 17%, 24%, 69%, 77%, 84%] of those studies showed that Prevadarin decreased the symptoms of the disorder.

3 A number of studies have been conducted to explore the impact of animal preserves on the poaching of apes and monkeys and [9%, 17%, 24%, 69%, 77%, 84%] of those studies showed that the preserves decrease the amount of poaching.

4 Astronomers using X-ray telescopes have analyzed the X-ray output of the sun and compared it to similar stars and [9%, 17%, 24%, 69%, 77%, 84%] of the studies confirmed that the sun has an X-ray output 30 times higher than similar stars.

5 Researchers at a number of universities have compared jaguar DNA to lynx DNA and [9%, 17%, 24%, 69%, 77%, 84%] found evidence that the lynx is the closest relative of the jaguar.

6 A number of geology papers on the geological history of Manhattan have been published and [9%, 17%, 24%, 69%, 77%, 84%] of them agree with the researchers at City College of New York that 34,000 years ago Manhattan was not an island but was part of the mainland.

7 A number of research teams at various universities have been studying

superconductivity and [9%, 17%, 24%, 69%, 77%, 84%] of those teams have produced evidence that magnesium diboride becomes a superconductor at -126 degrees Celsius.

8 Over the last decade, a series of scientific articles have been published on the effects of electrical stimulation of the splanchnic nerve and [9%, 17%, 24%, 69%, 77%, 84%] of those papers concluded that direct electrical stimulation of the splanchnic nerve does effectively treat cases of severe depression.

9 Dozens of scientific papers have been written on the cause of Lou Gehrig's disease and [9%, 17%, 24%, 69%, 77%, 84%] of those papers showed support for the hypothesis that clusters of prions in the brain cause Lou Gehrig's disease.

10 Several freshwater marine biologists have publicly weighed in on the fresh water perch issue and [9%, 17%, 24%, 69%, 77%, 84%] of them claim that snipe eels are the cause for the decline of the perch population.

11 Doctors at a number of hospitals have published their findings on the risks associated with laparoscopic surgery and [9%, 17%, 24%, 69%, 77%, 84%] found evidence to support the claim that laparoscopic surgery results in high levels of post operative infection when the technique is used to remove a person’s appendix.

12 Researchers in both Europe and the U.S. have been investigating Amgonalen and [9%, 17%, 24%, 69%, 77%, 84%] of those researchers have found support for the claim that Amgonalen effectively treats schizoid personality disorder.

Table S3. Additional Information for Experiment 1a, Negative Frame.

1 A number of research teams have looked for a connection between lutein and brain development and [91%, 83%, 76%, 31%, 23%, 16%] have failed to find a benefit for lutein.

2 Researchers at a number of universities tested Prevadarin for its effect on obsessive compulsive disorder and [91%, 83%, 76%, 31%, 23%, 16%] of those studies failed to show that Prevadarin decreased the symptoms of the disorder.

3 A number of studies have been conducted to explore the impact of animal preserves on the poaching of apes and monkeys and [91%, 83%, 76%, 31%, 23%, 16%] of those studies failed to show that the preserves decrease the amount of poaching.

4 Astronomers using X-ray telescopes have analyzed the X-ray output of the sun and compared it to similar stars and [91%, 83%, 76%, 31%, 23%, 16%] of the studies failed to confirm that the sun has an X-ray output 30 times higher than similar stars.

5 Researchers at a number of universities have compared jaguar DNA to lynx DNA and [91%, 83%, 76%, 31%, 23%, 16%] failed to find evidence that the lynx is the closest relative of the jaguar.

6 A number of geology papers on the geological history of Manhattan have been published and [91%, 83%, 76%, 31%, 23%, 16%] of them did not agree with the researchers at City College of New York that 34,000 years ago Manhattan was not an island but was part of the mainland.

7 A number of research teams at various universities have been studying superconductivity and [91%, 83%, 76%, 31%, 23%, 16%] of those teams have failed to produce evidence that magnesium diboride becomes a superconductor at -126 degrees Celsius.

8 Over the last decade, a series of scientific articles have been published on the effects of electrical stimulation of the splanchnic nerve and [91%, 83%, 76%, 31%, 23%, 16%] of those papers did not conclude that direct electrical stimulation of the splanchnic nerve does effectively treat cases of severe depression.

9 Dozens of scientific papers have been written on the cause of Lou Gehrig's disease and [91%, 83%, 76%, 31%, 23%, 16%] of those papers failed to show support for the hypothesis that clusters of prions in the brain cause Lou Gehrig's disease.

10 Several freshwater marine biologists have publicly weighed in on the fresh water perch issue and [91%, 83%, 76%, 31%, 23%, 16%] of them did not claim that snipe eels are the cause for the decline of the perch population.

11 Doctors at a number of hospitals have published their findings on the risks associated with laparoscopic surgery and [91%, 83%, 76%, 31%, 23%, 16%] failed to find evidence to support the claim that laparoscopic surgery results in high levels of post operative infection when the technique is used to remove a person’s appendix.

12 Researchers in both Europe and the U.S. have been investigating Amgonalen and [91%, 83%, 76%, 31%, 23%, 16%] of those researchers have failed to find support for the claim that Amgonalen effectively treats schizoid personality disorder.

Table S4. Additional Information for Experiment 3, Positive Frame.

1	Ten research teams have looked for a connection between lutein and brain development and [none, 2, 4, 6, 8, all] have found a benefit for lutein.
2	Researchers at a number of universities tested Prevadarin for its effect on obsessive compulsive disorder and [0, 2, 4, 6, 8, 10] out of 10 of those studies showed that Prevadarin decreased the symptoms of the disorder.
3	Ten studies have been conducted to explore the impact of animal preserves on the poaching of apes and monkeys and [none, 2, 4, 6, 8, all] of those studies showed that the preserves decrease the amount of poaching.
4	Astronomers using X-ray telescopes have analyzed the X-ray output of the sun and compared it to similar stars and [0, 2, 4, 6, 8, 10] out of 10 of the studies confirmed that the sun has an X-ray output 30 times higher than similar stars.
5	Researchers at a number of universities have compared jaguar DNA to lynx DNA and [0, 2, 4, 6, 8, 10] out of 10 found evidence that the lynx is the closest relative of the jaguar.
6	Ten geology papers on the geological history of Manhattan have been published and [none, 2, 4, 6, 8, all] of them agree with the researchers at City College of New York that 34,000 years ago Manhattan was not an island but was part of the mainland.
7	A number of research teams at various universities have been studying superconductivity and [0, 2, 4, 6, 8, 10] out of 10 of those teams have produced evidence that magnesium diboride becomes a superconductor at -126 degrees Celsius.

8 Over the last decade, 10 scientific articles have been published on the effects of electrical stimulation of the splanchnic nerve and [none, 2, 4, 6, 8, all] of those papers concluded that direct electrical stimulation of the splanchnic nerve effectively treats cases of severe depression.

9 Ten scientific papers have been written on the cause of Lou Gehrig's disease and [none, 2, 4, 6, 8, all] of those papers showed support for the hypothesis that clusters of prions in the brain cause Lou Gehrig's disease.

10 Several freshwater marine biologists have tried to replicate the study that showed that snipe eels are the cause for the decline of the perch population, and [0, 2, 4, 6, 8, 10] out of 10 of them found that snipe eels are the cause for the decline of the perch population.

11 Doctors at 10 hospitals have published their findings on the risks associated with laparoscopic surgery and [none, 2, 4, 6, 8, all] found evidence to support the claim that laparoscopic surgery results in high levels of post operative infection when the technique is used to remove a person’s appendix.

12 Researchers in both Europe and the U.S. have been investigating Amgonalen and [0, 2, 4, 6, 8, 10] out of 10 of those researchers have found support for the claim that Amgonalen effectively treats schizoid personality disorder.

Table S5. Additional Information for Experiment 3, Negative Frame.

1 Ten research teams have looked for a connection between lutein and brain development and [all, 8, 6, 4, 2, none] of them failed to find a benefit for lutein.

2 Researchers at a number of universities tested Prevadarin for its effect on obsessive compulsive disorder and [10, 8, 6, 4, 2, 0] out of 10 of those studies failed to show that Prevadarin decreased the symptoms of the disorder.

3 Ten studies have been conducted to explore the impact of animal preserves on the poaching of apes and monkeys and [all, 8, 6, 4, 2, none] of those studies failed to show that the preserves decrease the amount of poaching.

4 Astronomers using X-ray telescopes have analyzed the X-ray output of the sun and compared it to similar stars and [10, 8, 6, 4, 2, 0] out of 10 of the studies failed to confirm that the sun has an X-ray output 30 times higher than similar stars.

5 Researchers at a number of universities have compared jaguar DNA to lynx DNA and [10, 8, 6, 4, 2, 0] out of 10 failed to find evidence that the lynx is the closest relative of the jaguar.

6 Ten geology papers on the geological history of Manhattan have been published and [all, 8, 6, 4, 2, none] of them disagreed with the researchers at City College of New York that 34,000 years ago Manhattan was not an island but was part of the mainland.

7 A number of research teams at various universities have been studying superconductivity and [10, 8, 6, 4, 2, 0] out of 10 of those teams have failed to produce evidence that magnesium diboride becomes a superconductor at -126

degrees Celsius.

8 Over the last decade, 10 scientific articles have been published on the effects of electrical stimulation of the splanchnic nerve and [10, 8, 6, 4, 2, 0] of those papers did not conclude that direct electrical stimulation of the splanchnic nerve effectively treats cases of severe depression.

9 Ten scientific papers have been written on the cause of Lou Gehrig's disease and [all, 8, 6, 4, 2, none] of those papers failed to show support for the hypothesis that clusters of prions in the brain cause Lou Gehrig's disease.

10 Several freshwater marine biologists have tried to replicate the study that showed that snipe eels are the cause for the decline of the perch population, and [10, 8, 6, 4, 2, 0] out of 10 of them failed to find that snipe eels are the cause for the decline of the perch population.

11 Doctors at 10 hospitals have published their findings on the risks associated with laparoscopic surgery and [all, 8, 6, 4, 2, none] failed to find evidence to support the claim that laparoscopic surgery results in high levels of post operative infection when the technique is used to remove a person’s appendix.

12 Researchers in both Europe and the U.S. have been investigating Amgonalen and [10, 8, 6, 4, 2, 0] out of 10 of those researchers failed to find support for the claim that Amgonalen effectively treats schizoid personality disorder.

Note. For item 6, statements with 0 of 10 and 10 of 10 used “disagreed” instead of “failed to agree” in order to avoid awkward phrasing. “Failed to agree was used for all other statements, mirroring the stimuli in Experiments 1 and 2.

Table S6. Experiment 5 stimuli

**Replication Rate**	**Frame**	**Additional information**
8%	Positive	Additionally, 8% of the researchers who tried to replicate Dr. Simonson's findings succeeded in doing so.
	Negative	Additionally, 92% of the researchers who tried to replicate Dr. Simonson's findings failed to do so.
43%	Positive	Additionally, 43% of the researchers who tried to replicate Dr. Simonson's findings succeeded in doing so.
	Negative	Additionally, 57% of the researchers who tried to replicate Dr. Simonson's findings failed to do so.
81%	Positive	Additionally, 81% of the researchers who tried to replicate Dr. Simonson's findings succeeded in doing so.
	Negative	Additionally, 19% of the researchers who tried to replicate Dr. Simonson's findings failed to do so.
92%	Positive	Additionally, 92% of the researchers who tried to replicate Dr. Simonson's findings succeeded in doing so.
	Negative	Additionally, 8% of the researchers who tried to replicate Dr. Simonson's findings failed to do so.
4 of 10	Positive	Additionally, 4 out of 10 of the researchers who tried to replicate Dr. Simonson's findings succeeded in doing so.
	Negative	Additionally, 6 out of 10 of the researchers who tried to replicate Dr. Simonson's findings failed to do so.
2 of 5	Positive	Additionally, 2 out of 5 of the researchers who tried to replicate Dr. Simonson's findings succeeded in doing so.
	Negative	Additionally, 3 out of 5 of the researchers who tried to replicate Dr. Simonson's findings failed to do so.

Table S7. Summary of sequence of items within each questionnaire.*

Experiment	Questionnaire	Sequence of 24 items
1A	1	distractor1, topic9, topic8_9%, topic7, distractor2, distractor3, distractor4, topic10_77%, topic12_84%, topic3, distractor5, topic1, distractor6, topic2_17%, distractor7, topic6_69%, topic5, distractor8, distractor9, topic4_24%, distractor10, topic11, distractor11, distractor12
	2	distractor1, topic9_9%, topic8, topic7_69%, distractor2, distractor3, distractor4, topic10, topic12, topic3_17%, distractor5, topic1_84%, distractor6, topic2, distractor7, topic6, topic5_24%, distractor8, distractor9, topic4, distractor10, topic11_77%, distractor11, distractor12
	3	distractor1, topic9, topic8_69%, topic7, distractor2, distractor3, distractor4, topic10_17%, topic12_24%, topic3, distractor5, topic1, distractor6, topic2_77%, distractor7, topic6_9%, topic5, distractor8, distractor9, topic4_84%, distractor10, topic11, distractor11, distractor12
	4	distractor1, topic9_69%, topic8, topic7_9%, distractor2, distractor3, distractor4, topic10, topic12, topic3_77%, distractor5, topic1_24%, distractor6, topic2, distractor7, topic6, topic5_84%, distractor8, distractor9, topic4, distractor10, topic11_17%, distractor11, distractor12
	5	distractor1, topic9, topic8_17%, topic7, distractor2, distractor3, distractor4, topic10_84%, topic12_69%, topic3, distractor5, topic1, distractor6, topic2_24%, distractor7, topic6_77%, topic5, distractor8, distractor9, topic4_9%, distractor10, topic11, distractor11, distractor12
	6	distractor1, topic9_17%, topic8, topic7_77%, distractor2, distractor3, distractor4, topic10, topic12, topic3_24%, distractor5, topic1_69%, distractor6, topic2, distractor7, topic6, topic5_9%, distractor8, distractor9, topic4, distractor10, topic11_84%, distractor11, distractor12
	7	distractor1, topic9, topic8_77%, topic7, distractor2, distractor3, distractor4, topic10_24%, topic12_9%, topic3, distractor5, topic1, distractor6, topic2_84%, distractor7, topic6_17%, topic5, distractor8, distractor9, topic4_69%, distractor10, topic11, distractor11, distractor12
	8	distractor1, topic9_77%, topic8, topic7_17%, distractor2, distractor3, distractor4, topic10, topic12, topic3_84%, distractor5, topic1_9%, distractor6, topic2, distractor7, topic6, topic5_69%, distractor8, distractor9, topic4, distractor10, topic11_24%, distractor11, distractor12

1B	1	distractor1, topic9, topic8_97%, topic7, distractor2, distractor3, distractor4, topic10_32%, topic12_4%, topic3, distractor5, distractor6, distractor7, topic2_92%, distractor8, topic6_42%, topic5, topic1, distractor9, topic4_54%, distractor10, topic11, distractor11, distractor12
	2	distractor1, topic9_97%, topic8, topic7_42%, distractor2, distractor3, distractor4, topic10, topic12, topic3_92%, distractor5, distractor6, distractor7, topic2, distractor8, topic6, topic5_54%, topic1_4%, distractor9, topic4, distractor10, topic11_32%, distractor11, distractor12
	3	distractor1, topic9, topic8_42%, topic7, distractor2, distractor3, distractor4, topic10_92%, topic12_54%, topic3, distractor5, distractor6, distractor7, topic2_32%, distractor8, topic6_97%, topic5, topic1, distractor9, tpic4_4%, distractor10, topic11, distractor11, distractor12
	4	distractor1, topic9_42%, topic8, topic7_97%, distractor2, distractor3, distractor4, topic10, topic12, topic3_32%, distractor5, distractor6, distractor7, topic2, distractor8, topic6, topic5_4%, topic1_69%, distractor9, topic4, distractor10, topic11_92%, distractor11, distractor12
	5	distractor1, topic9, topic8_92%, topic7, distractor2, distractor3, distractor4, topic10_4%, topic12_42%, topic3, distractor5, distractor6, distractor7, topic2_54%, distractor8, topic6_32%, topic5, topic1, distractor9, topic4_97%, distractor10, topic11, distractor11, distractor12
	6	distractor1, topic9_92%, topic8, topic7_32%, distractor2, distractor3, distractor4, topic10, topic12, topic3_54%, distractor5, distractor6, distractor7, topic2, distractor8, topic6, topic5_97%, topic1_42%, distractor9, topic4, distractor10, topic11_4%, distractor11, distractor12
	7	distractor1, topic9, topic8_32%, topic7, distractor2, distractor3, distractor4, topic10_54%, topic12_97%, topic3, distractor5, distractor6, distractor7, topic2_4%, distractor8, topic6_92%, topic5, topic1, distractor9, topic4_42%, distractor10, topic11, distractor11, distractor12
	8	distractor1, topic9_32%, topic8, topic7_92%, distractor2, distractor3, distractor4, topic10, topic12, topic3_4%, distractor5, distractor6, distractor7, topic2, distractor8, topic6, topic5_42%, topic1_97%, distractor9, topic4, distractor10, topic11_54%, distractor11, distractor12

2	1	distractor1, topic9, topic8_97%, topic7, distractor2, distractor3, distractor4, topic10_17%, topic12_4%, topic3, distractor5, distractor6, distractor7, topic2_84%, distractor8, topic6_42%, topic5, topic1, distractor9, topic4_69%, distractor10, topic11, distractor11, distractor12
	2	distractor1, topic9_97%, topic8, topic7_42%, distractor2, distractor3, distractor4, topic10, topic12, topic3_84%, distractor5, distractor6, distractor7, topic2, distractor8, topic6, topic5_69%, topic1_4%, distractor9, topic4, distractor10, topic11_17%, distractor11, distractor12
	3	distractor1, topic9, topic8_42%, topic7, distractor2, distractor3, distractor4, topic10_84%, topic12_69%, topic3, distractor5, distractor6, distractor7, topic2_17%, distractor8, topic6_97%, topic5, topic1, distractor9, tpic4_4%, distractor10, topic11, distractor11, distractor12
	4	distractor1, topic9_42%, topic8, topic7_97%, distractor2, distractor3, distractor4, topic10, topic12, topic3_17%, distractor5, distractor6, distractor7, topic2, distractor8, topic6, topic5_4%, topic1_69%, distractor9, topic4, distractor10, topic11_84%, distractor11, distractor12
	5	distractor1, topic9, topic8_84%, topic7, distractor2, distractor3, distractor4, topic10_4%, topic12_42%, topic3, distractor5, distractor6, distractor7, topic2_69%, distractor8, topic6_17%, topic5, topic1, distractor9, topic4_97%, distractor10, topic11, distractor11, distractor12
	6	distractor1, topic9_84%, topic8, topic7_17%, distractor2, distractor3, distractor4, topic10, topic12, topic3_69%, distractor5, distractor6, distractor7, topic2, distractor8, topic6, topic5_97%, topic1_42%, distractor9, topic4, distractor10, topic11_4%, distractor11, distractor12
	7	distractor1, topic9, topic8_17%, topic7, distractor2, distractor3, distractor4, topic10_69%, topic12_97%, topic3, distractor5, distractor6, distractor7, topic2_4%, distractor8, topic6_84%, topic5, topic1, distractor9, topic4_42%, distractor10, topic11, distractor11, distractor12
	8	distractor1, topic9_17%, topic8, topic7_84%, distractor2, distractor3, distractor4, topic10, topic12, topic3_4%, distractor5, distractor6, distractor7, topic2, distractor8, topic6, topic5_42%, topic1_97%, distractor9, topic4, distractor10, topic11_69%, distractor11, distractor12

3	1	distractor1, topic9, topic8_0, topic7, distractor2, distractor3, distractor4, topic10_8, topic12_10, topic3, distractor5, topic1, distractor6, topic2_2, distractor7, topic6_6, topic5, distractor8, distractor9, topic4_4, distractor10, topic11, distractor11, distractor12
	2	distractor1, topic9_0, topic8, topic7_6, distractor2, distractor3, distractor4, topic10, topic12, topic3_2, distractor5, topic1_10, distractor6, topic2, distractor7, topic6, topic5_4, distractor8, distractor9, topic4, distractor10, topic11_8, distractor11, distractor12
	3	distractor1, topic9, topic8_6, topic7, distractor2, distractor3, distractor4, topic10_2, topic12_4, topic3, distractor5, topic1, distractor6, topic2_8, distractor7, topic6_0, topic5, distractor8, distractor9, topic4_10, distractor10, topic11, distractor11, distractor12
	4	distractor1, topic9_6, topic8, topic7_0, distractor2, distractor3, distractor4, topic10, topic12, topic3_8, distractor5, topic1_4, distractor6, topic2, distractor7, topic6, topic5_10, distractor8, distractor9, topic4, distractor10, topic11_2, distractor11, distractor12
	5	distractor1, topic9, topic8_2, topic7, distractor2, distractor3, distractor4, topic10_10, topic12_6, topic3, distractor5, topic1, distractor6, topic2_4, distractor7, topic6_8, topic5, distractor8, distractor9, topic4_0, distractor10, topic11, distractor11, distractor12
	6	distractor1, topic9_2, topic8, topic7_8, distractor2, distractor3, distractor4, topic10, topic12, topic3_4, distractor5, topic1_6, distractor6, topic2, distractor7, topic6, topic5_0 distractor8, distractor9, topic4, distractor10, topic11_10, distractor11, distractor12
	7	distractor1, topic9, topic8_8, topic7, distractor2, distractor3, distractor4, topic10_4, topic12_0, topic3, distractor5, topic1, distractor6, topic2_10, distractor7, topic6_2, topic5, distractor8, distractor9, topic4_6, distractor10, topic11, distractor11, distractor12
	8	distractor1, topic9_8, topic8, topic7_2, distractor2, distractor3, distractor4, topic10, topic12, topic3_10, distractor5, topic1_0, distractor6, topic2, distractor7, topic6, topic5_6, distractor8, distractor9, topic4, distractor10, topic11_4, distractor11, distractor12

4	1	distractor1, topic9, topic8_0, topic7, distractor2, distractor3, distractor4, topic10_4, topic12_5, topic3, distractor5, topic1, distractor6, topic2_1, distractor7, topic6_3, topic5, distractor8, distractor9, topic4_2, distractor10, topic11, distractor11, distractor12
	2	distractor1, topic9_0, topic8, topic7_3, distractor2, distractor3, distractor4, topic10, topic12, topic3_1, distractor5, topic1_5, distractor6, topic2, distractor7, topic6, topic5_2, distractor8, distractor9, topic4, distractor10, topic11_4, distractor11, distractor12
	3	distractor1, topic9, topic8_3, topic7, distractor2, distractor3, distractor4, topic10_1, topic12_2, topic3, distractor5, topic1, distractor6, topic2_4, distractor7, topic6_0, topic5, distractor8, distractor9, topic4_5, distractor10, topic11, distractor11, distractor12
	4	distractor1, topic9_3, topic8, topic7_0, distractor2, distractor3, distractor4, topic10, topic12, topic3_4, distractor5, topic1_2, distractor6, topic2, distractor7, topic6, topic5_5, distractor8, distractor9, topic4, distractor10, topic11_1, distractor11, distractor12
	5	distractor1, topic9, topic8_1, topic7, distractor2, distractor3, distractor4, topic10_5, topic12_3, topic3, distractor5, topic1, distractor6, topic2_2, distractor7, topic6_4, topic5, distractor8, distractor9, topic4_0, distractor10, topic11, distractor11, distractor12
	6	distractor1, topic9_1, topic8, topic7_4, distractor2, distractor3, distractor4, topic10, topic12, topic3_2, distractor5, topic1_3, distractor6, topic2, distractor7, topic6, topic5_0 distractor8, distractor9, topic4, distractor10, topic11_5, distractor11, distractor12
	7	distractor1, topic9, topic8_4, topic7, distractor2, distractor3, distractor4, topic10_2, topic12_0, topic3, distractor5, topic1, distractor6, topic2_5, distractor7, topic6_1, topic5, distractor8, distractor9, topic4_3, distractor10, topic11, distractor11, distractor12
	8	distractor1, topic9_4, topic8, topic7_1, distractor2, distractor3, distractor4, topic10, topic12, topic3_5, distractor5, topic1_0, distractor6, topic2, distractor7, topic6, topic5_3, distractor8, distractor9, topic4, distractor10, topic11_2, distractor11, distractor12

*Each questionnaire contained 24 items. Twelve items were distractors, 12 were critical items. Critical items could be science claims in isolation or science claims paired with replication rate information. When present, replication rate information is found after the underscore "_" character. Only positive frame questionnaires are presented. Negative frame questionnaires were identical in terms of sequence except for the change in frame.

Stimuli S1. Questionnaire 1 from Experiment 1a

Instructions

For each issue, please *carefully* read all the material presented to you. The paragraph on the left will promote a particular hypothesis. The paragraph on the right (if present) will present additionally information. After you have read the material for a particular issue, indicate your feelings about the way you feel about the hypothesis promoted in the paragraph on the ***left.***

Example issue: the cause of the common cold

Claim A

According to one source…<br>The common cold is caused by a virus.	*According to another source…*<br>The common cold is caused by going outside in the winter with wet hair.

Favor Claim A 1 (2) 3 4 5 6 Oppose Claim A

In the example above you would circle a
1 if you strongly favored the virus hypothesis
2 if you moderately favored the virus hypothesis
3 if you somewhat favored the virus hypothesis
4 if you somewhat opposed the virus hypothesis
5 if you moderately opposed the virus hypothesis
6 if you strongly opposed the virus hypothesis

In the example above, the "2" is circled indicating that the person moderately favored the virus hypothesis.

You should not skip any issues. Please circle a number in the scale below *each* issue.

Section 1

Claim #1
According to one source…
According to Dr. Hargrave of Haskins Engineering Lab, cars powered by hybrid engines will always use less fossil fuel than those powered by fuel cells.

**Favor Claim #1**	**1**	**2**	**3**	**4**	**5**	**6**	**Oppose Claim #1**

Claim #2
According to one source…
Clusters of prions in the brain cause Lou Gehrig's disease.

**Favor Claim #2**	**1**	**2**	**3**	**4**	**5**	**6**	**Oppose Claim #2**

Claim #3
According to one source…
Direct electrical stimulation of the splanchnic nerve is effective at treating cases of severe depression.

According to another source…
Over the last decade, a series of scientific articles have been published on the effects of electrical stimulation of the splanchnic nerve and 9% of those papers concluded that direct electrical stimulation of the splanchnic nerve does effectively treat cases of severe depression.

**Favor Claim #3**	**1**	**2**	**3**	**4**	**5**	**6**	**Oppose Claim #3**

Claim #4
According to one source…
Magnesium diboride becomes a superconductor at -126 degrees Celsius.

Favor Claim #4 1 2 3 4 5 6 Oppose Claim #4

Claim #5
According to one source…
Dr. Zuirette of Brown University claims that the plastic used in fast food cups contains a chemical called oxalicide which has been recently linked with laryngeal cancer.

.

Favor Claim #5 1 2 3 4 5 6 Oppose Claim #5

Claim #6
According to one source…
According to Dr. Johnson from the American Dermatological Association, Banana Boat brand tanning lotion blocks more UVA and UVB rays than the lotions made by their competitors.

According to another source…
Dr. Johnson received her advanced degree from a university with a reputation for having very low standards.

Favor Claim #6 1 2 3 4 5 6 Oppose Claim #6

Claim #7

According to one source…
Dr. Jensen, a botanist at the University of California, claims that the chemicals used in the Roundup brand herbicide are completely harmless to children.

According to another source…
Dr. Jensen is head of the research and development branch of the company that manufactures Roundup herbicide.

Favor Claim #7 1 2 3 4 5 6 Oppose Claim #7

Claim #8

According to one source…
The decline of the population of fresh water perch in Lake Erie is due to competition from snipe eels, a non-native species that was introduced to the Lake in the 1990s.

According to another source…
Several freshwater marine biologists have publicly weighed in on the fresh water perch issue and 77% of them claim that snipe eels are the cause for the decline of the perch population.

Favor Claim #8 1 2 3 4 5 6 Oppose Claim #8

Claim #9

According to one source…
A new drug, Amgonalen, effectively treats schizoid personality disorder.

According to another source…
Researchers in both Europe and the U.S. have been investigating Amgonalen and 84% of those researchers have found support for the claim that Amgonalen effectively treats schizoid personality disorder.

Favor Claim #9 1 2 3 4 5 6 Oppose Claim #9

Claim #10
According to one source…
A team of African biologists found that creating a series of medium sized animal preserves throughout a rainforest drastically decreases the poaching of apes and monkeys by hunters.

Favor Claim #10 1 2 3 4 5 6 Oppose Claim #10

Claim #11
According to one source…
For the last couple of years, whale populations have been in sharp decline. According to marine biologist Dr. Rose, one reason why whale populations have been decreasing at such high rates is because the ultra high intensity sonar recently installed on US submarines is blasting the ear drums of whales so that they cannot hear. If whales can't find each other by sound, then they cannot mate with each other.

Favor Claim #11 1 2 3 4 5 6 Oppose Claim #11

Claim #12
According to one source…
According to a research team at U.C.L.A., infants that consumed formula that was supplemented with lutein had higher I.Q. scores at age 10 compared to infants that consumed regular formula. Therefore, the nutritional supplement lutein, when given to infants enhances brain development.

Favor Claim #12 1 2 3 4 5 6 Oppose Claim #12

Claim #13

According to one source…
According to Dr. Cho's research, small amounts of the plastic in the plastic bottles used by some baby food companies leaches into the baby food. She claims that this plastic can act a carcinogen and that it is dangerous to children.

According to another source…
Many of the researchers in Dr. Cho's field feel that she is a sloppy researcher.

Favor Claim #13 1 2 3 4 5 6 Oppose Claim #13

Claim #14

According to one source…
According to two prominent psychiatrists, a new drug, Prevadarin, effectively treats obsessive compulsive disorder.

According to another source…
Researchers at a number of universities tested Prevadarin for its effect on obsessive compulsive disorder and 17% of those studies showed that Prevadarin decreased the symptoms of the disorder.

Favor Claim #14 1 2 3 4 5 6 Oppose Claim #14

Claim #15

According to one source…
Dr. Gray from New England Medical Center recently disclosed research findings that indicate that the consumption of redfish leads to an increased risk of lymphoblastic leukemia.

Favor Claim #15 1 2 3 4 5 6 Oppose Claim #15

Claim #16

According to one source…
A team of researchers at City College of New York concluded from the rock samples they analyzed that 34,000 years ago Manhattan was not an island but was part of the mainland.

According to another source…
A number of geology papers on the geological history of Manhattan have been published and 69% of them agree with the researchers at City College of New York that 34,000 years ago Manhattan was not an island but was part of the mainland.

Favor Claim #16 **1** **2** **3** **4** **5** **6** **Oppose Claim #16**

Claim #17

According to one source…
A group of biologists at Washington State University recently compared the DNA of jaguars with that of other great cats. They found the highest degree of similarity between jaguars and lynxes and concluded that the lynx is the closest relative of the jaguar.

Favor Claim #17 **1** **2** **3** **4** **5** **6** **Oppose Claim #17**

Claim #18

According to one source…
Dr. Anderson of Timkin Labs has shown that steel brake pads doped with small amounts of nickel wear out faster than pads that are not doped with nickel.

According to another source…
While it is true that, in theory, doping steel with nickel will make it wear out faster, it is also true that adding nickel prevents the brakes from rusting. Dr. Anderson failed to take the issue of rust into account.

Favor Claim #18 **1** **2** **3** **4** **5** **6** **Oppose Claim #18**

Claim #19

According to one source…
Dr. Doyle from the Children's Hospital of Pittsburgh claims that the chances of a child being diagnosed with Prudar-Wein syndrome decrease by over 20% if their diet includes niacin enriched baby food.

According to another source…
Recently a team of investigators from the National Science Foundation's ethics committee found that Dr. Doyle fabricated some of the data in one of her earlier papers.

Favor Claim #19 1 2 3 4 5 6 Oppose Claim #19

Claim #20

According to one source…
A team of astronomers at the Chandra X-ray Observatory found that the sun emits 30 times more X-rays than most other stars of the same size and age.

According to another source…
Astronomers using X-ray telescopes have analyzed the X-ray output of the sun and compared it to similar stars and 24% of the studies confirmed that the sun has an X-ray output 30 times higher than similar stars.

Favor Claim #20 1 2 3 4 5 6 Oppose Claim #20

Claim #21

According to one source…
According to Dr. Martinez at the University of Oklahoma, dibutylphthalate, a chemical used in Gold Bond foot powder, decreases the risk of some kinds of cancer.

Favor Claim #21 1 2 3 4 5 6 Oppose Claim #21

Claim #22
According to one source…
Laparoscopic surgery results in high levels of post operative infection when the technique is used to remove a person's appendix.

Favor Claim #22 1 2 3 4 5 6 Oppose Claim #22

Claim #23
According to one source…
According to Dr. Smith, a climate and energy researcher, nuclear power is just as inexpensive as power from coal and nuclear power has zero CO2 emissions.

Favor Claim #23 1 2 3 4 5 6 Oppose Claim #23

Claim #24
According to one source…
According to Dr. Gumbilo and her group of ecologists at the University of Ohio, plane travel causes more global warming than car travel.

According to another source…
Recently a team of investigators from the National Science Foundation's ethics committee found that Dr. Gumbilo fabricated some of the data in her published research on global warming.

Favor Claim #24 1 2 3 4 5 6 Oppose Claim #24

12a

Section 2

1. How would you describe the preceding paragraphs? (circle all that apply)
    a. Irritating
    b. Confusing
    c. Very technical
    d. Boring
    e. Interesting
    f. Fun
    g. None of the above

2. How knowledgeable would you say that you are in regards to biological and medical science?
    a. Not very knowledgeable
    b. Somewhat knowledgeable
    c. Moderately knowledgeable
    d. Very knowledgeable

3. How knowledgeable would you say you are in regards to chemistry and physics?
    a. Not very knowledgeable
    b. Somewhat knowledgeable
    c. Moderately knowledgeable
    d. Very knowledgeable

4. What is your opinion about the scientific organizations mentioned in the preceding paragraphs?
    a. Little faith should be placed in the claims of these organizations
    b. Moderate faith should be placed in the claims of these organizations
    c. A great deal of faith should be placed in the claims of these organizations
    d. I do not know if I should place faith in the claims of these organizations

5. Did you read both paragraphs for each item or did you occasionally just read one paragraph and not the other? *(remember you can be honest because your answers will be anonymous)*
    a. Always read both paragraphs for each issue
    b. Occasionally just read one paragraph
    c. Usually just read one paragraph

6. Did you give serious and thoughtful answers to all the items or did you just make responses without thinking about the issues so that you could get the experiment over with? *(remember you can be honest because your answers will be anonymous)*
    a. Always gave serious and thoughtful answers
    b. Occasionally just circled a number without thinking
    c. Usually just circled a number without thinking

Section 2

Sex (circle one)
F M

Age

_____

Field of study (actual or intended)

a. Natural science (e.g. physics, chemistry, biology, etc.)
b. Social science (e.g. psychology, sociology, political science, etc.)
c. Other (e.g. languages, religion, math, business, nursing, etc.)

Class status

a. College freshman
b. College sophomore
c. College junior
d. College senior
e. Bachelor’s degree (or more) completed

If applicable, what is the course title for which you are completing this survey?
(e.g. Introduction to Psychology, Methods & statistics, etc.)

____

University or college attended _________________
